# Root Exudate Chemotypes and Soil Improvement in Orchard Green Manures: Species-Specific Associations Reflecting Family-Level Phylogenetic Differences

**DOI:** 10.3390/plants15203190

**Published:** 2026-10-18

**Authors:** Minghao Sun, Xing Shen, Yongjie Liu, Wenyu Chen, Zhongping Chai

**Affiliations:** 1 College of Resources and Environment, Xinjiang Agricultural University, Urumqi 830052, China; sun18453303381@126.com (M.S.); chenwenyu666666@163.com (W.C.); chaizhongpingth@sina.com (Z.C.); 2 Korla Fragrant Pear Research Center, Bayingolin Mongol Autonomous Prefecture, Korla 841000, China

**Keywords:** root exudates, rhizosphere effect, green manure, chemotype, temporal dynamics

## Abstract

Green manures differ widely in their capacity to modify the rhizosphere soil environment. However, the link between root exudate chemistry and changes in soil properties remains poorly understood. We grew alfalfa (*Medicago sativa* L.), white clover (*Trifolium repens* L.), smooth bromegrass (*Bromus inermis* Leyss.) and perennial ryegrass (*Lolium perenne* L.) in pear orchard inter-rows, sampling rhizosphere soil (operationally defined here as the 1–4 mm root-adhering layer) and inter-row non-rhizosphere background soil at four stages within a single growing season. Root exudates (112 compounds) were profiled by GC-MS and analyzed with soil properties using compositional data approaches. The four species formed distinguishable chemotypes. Rhizosphere effects diverged: perennial ryegrass showed the largest relative increase in extractable P versus its own inter-row non-rhizosphere background (+37.0%), whereas absolute rhizosphere available P was highest in alfalfa (48.3 mg kg^−1^), which also accumulated the most nitrogen (total N +19.6%), and white clover buffered pH best. This increase in extractable P was accompanied by lower rhizosphere TN and AN and does not by itself demonstrate enhanced *p* acquisition, which in calcareous soils depends on sorption and Ca-phosphate precipitation. PCA separated species along phosphorus–organic, matter–salinity, and nitrogen–potassium gradients (PERMANOVA R^2^ = 0.365, *p* = 0.001). After center log-ratio (CLR) correction, the chemotype remained coupled to soil properties (Mantel *r* = 0.246, *p* = 0.005), but fell to *r* = 0.062 (*p* = 0.236) when family was controlled, indicating that the coupling was largely attributable to family-level background (two legumes versus two grasses). Among compound classes, amino acids showed the strongest coupling with the soil matrix (*r* = 0.32, *p* < 0.01). Among individual compounds, tryptophan showed the strongest uncorrected correlation with rhizosphere nitrogen (r = +0.83), although this association was not significant after multiple-testing correction (minimum q = 0.056) and should only be used for hypothesis generation purposes. Entropy-weighted TOPSIS and gray relational analysis (GRA) anchored to nitrogen function ranked alfalfa first in all scenarios, its lead robust to index composition. Overall, these findings are exploratory: the chemotype–soil association reflects family-structured multivariate covariation rather than single-molecule causation, and alfalfa is a tentative recommendation for this nitrogen-limited orchard, pending validation by controlled causal experiments.

## 1. Introduction

Orchard soils underpin the sustained productivity of perennial fruit trees, and microscale changes in their physicochemical properties can cascade through the entire root zone. Nutrient cycling and the soil microbial community influence this cascade through distinct pathways: nutrient cycling processes determine the chemical forms and supply rates of soil nutrients, whereas the microbial community drives mineralization, immobilization, nitrification, and phosphorus solubilization; plant growth responds directly to the resulting available nutrient pool, which is jointly but differently shaped by these two pathways. Unlike annual croplands, fruit trees occupy the soil profile throughout the year; rhizosphere processes are therefore persistent and spatially heterogeneous, and root-derived chemical signals spread outward from the root surface through root penetration and diffusion in the soil solution, improving the surrounding inter-row bulk soil as a whole. Korla fragrant pear (*Pyrus sinkiangensis*), a pillar industry of Xinjiang’s characteristic fruit sector, is increasingly affected by long-term clean tillage, which has depleted soil organic matter (OM), concentrated nutrients at the surface, and reduced biological activity [1,2]. Growing grass or green manures in the inter-rows has become an important agronomic practice for improving orchard soil structure and nutrient supply [1,3,4,5,6]. However, most studies of orchard green manures have focused on aboveground cover effects (biomass, weed suppression and moisture conservation) or on responses of single nutrient indices. The systematic linkage between rhizosphere exudation and changes in soil physicochemical properties remains insufficiently understood [7,8]. Green manure selection consequently remains largely empirical, with limited understanding of how species-specific rhizosphere functions translate into soil improvement outcomes, and precision configuration based on root–soil interactions is lacking.

Advances in metabolomics offer new tools for resolving this issue. High-throughput profiling by GC-MS now permits simultaneous detection of more than one hundred low-molecular-weight metabolites [9,10], showing that root exudation is not random chemical leakage but a structured output, the chemotype, under strict genetic control [11,12,13]. Existing evidence indicates that root exudates form a low-molecular-weight chemical matrix with marked species dependence [14,15], but most studies remain at the level of descriptive classification and do not ask how chemotypes map onto soil function. We know what different species secrete, but not how these differentiated chemical outputs are converted into differentiated soil improvement. A key unresolved question is whether compound classes as a whole drive the outcome, or whether a few high-abundance marker molecules carry the functional signal. This distinction is a prerequisite for building a predictive chemotype–soil response model. In this study, the chemotype is defined as the species-specific compositional pattern of mean relative abundances of seven compound classes across four developmental stages. It should be emphasized that detecting a large number of low-molecular-weight root releases does not by itself explain nutrient mobilization. In calcareous soils such as that of the study orchard, nutrient availability is governed by sorption and desorption equilibria, dissolution and re-precipitation of Ca-phosphates, pH buffering, ligand exchange, cation exchange and microbial mineralization and immobilization [16,17,18]. We therefore do not treat the detection of exudate compounds as evidence of nutrient mobilization; instead, the measured soil properties are interpreted as the net outcome of these soil chemical and biological processes.

Soil responses are themselves systemic: pH is coupled to phosphorus availability [16], organic matter inputs modify cation exchange capacity, and organic and phenolic acids both chelate metal ions and serve as microbial carbon sources [14]. No single index can therefore capture rhizosphere improvement, and multivariate methods are required to describe the overall pattern of variation. In addition, exudate composition fluctuates strongly with developmental stage [19,20], and whether such temporal variation corresponds to a stage-specific shift in the soil improvement trajectory remains empirically untested. These systemic and temporal dimensions have long been overlooked in orchard green manure research: most experiments compare mean differences among species or focus on a single stage, and therefore cannot reveal the coupling between exudate dynamics and the soil response trajectory, nor judge whether the rhizosphere effect is a simple sum of independent trait changes or an integrated output of coordinated responses [21].

We address three progressive questions using four functionally contrasting green manure species grown in the inter-rows of a Korla fragrant pear orchard, namely the legumes *Ms* and *Tr* and the grasses *Bi* and *Lp*. These four species are the most widely used green manures in Xinjiang orchards: they tolerate arid and saline–alkaline conditions, are easy to establish and manage, and together form a balanced comparison of two functionally contrasting families—two nitrogen-fixing legumes and two fibrous-rooted grasses. Rhizosphere soil, inter-row non-rhizosphere soil, and root exudates were sampled synchronously at four key developmental stages and analyzed by GC-MS metabolomics integrated with soil physicochemical properties. The first question asks whether the species express distinct and temporally dynamic exudate chemotypes that correspond to species differences in exudation profiles and metabolic strategies rather than representing random chemical mixtures [22,23,24]. The second asks whether chemotype differences correspond to systematic divergence of multiple rhizosphere soil traits that can be captured by multivariate ordination. The third examines whether compound classes and specific marker compounds fulfil hierarchical roles of strategic orientation and executive fine-tuning; that is, whether class-level flux matches the overall direction of soil change while marker molecules participate in fine regulation. On this basis, semi-quantification with an internal standard and multi-criteria evaluation (entropy-weighted TOPSIS and gray relational analysis) were used to translate the chemotype–soil evidence into a green manure recommendation for a nitrogen-limited orchard, providing a predictive framework for rhizosphere function-based evaluation and precision configuration of orchard green manures. Throughout this paper, the term ‘phylogenetic’ in the title refers to the conceptual perspective of the study—testing whether root exudate chemotypes and their soil effects differ between representative species of the two families—rather than a claim to have demonstrated a general phylogenetic law; all inferences are strictly limited to the four species studied.

## 2. Results

**Figure 1:**
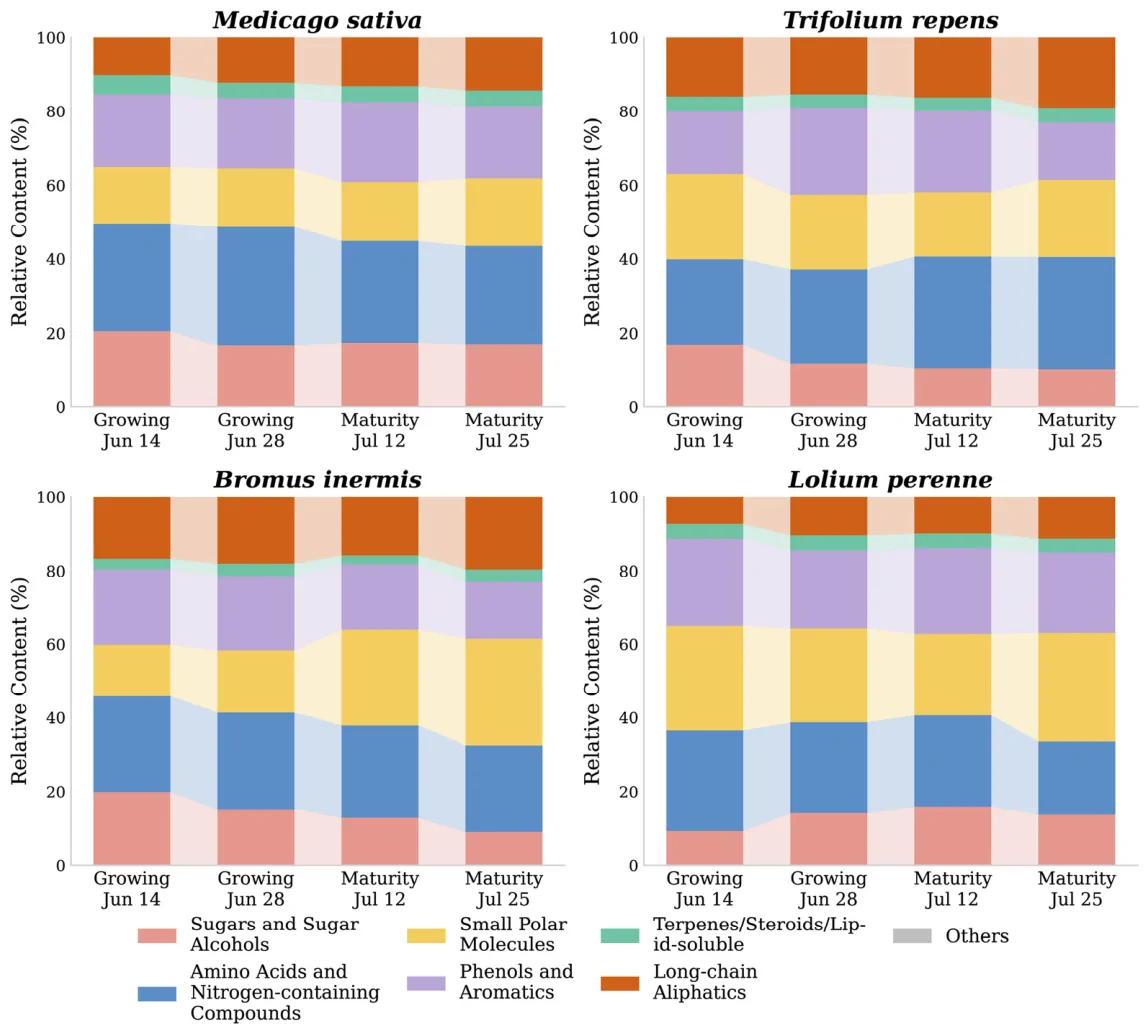
Temporal dynamics of the relative contents of seven exudate classes in the four green manures (stacked percentage bars by species, with color bands between adjacent stages showing gains and losses of each class; normalized to 100% per batch, *n* = 3; stage labels correspond to the BBCH-coded sampling dates defined in Section 4.1). Figure 1 is descriptive: all inferential statistics were computed from the underlying class abundance matrices (Section 4.4), not from the plotted percentages.

**Figure 2:**
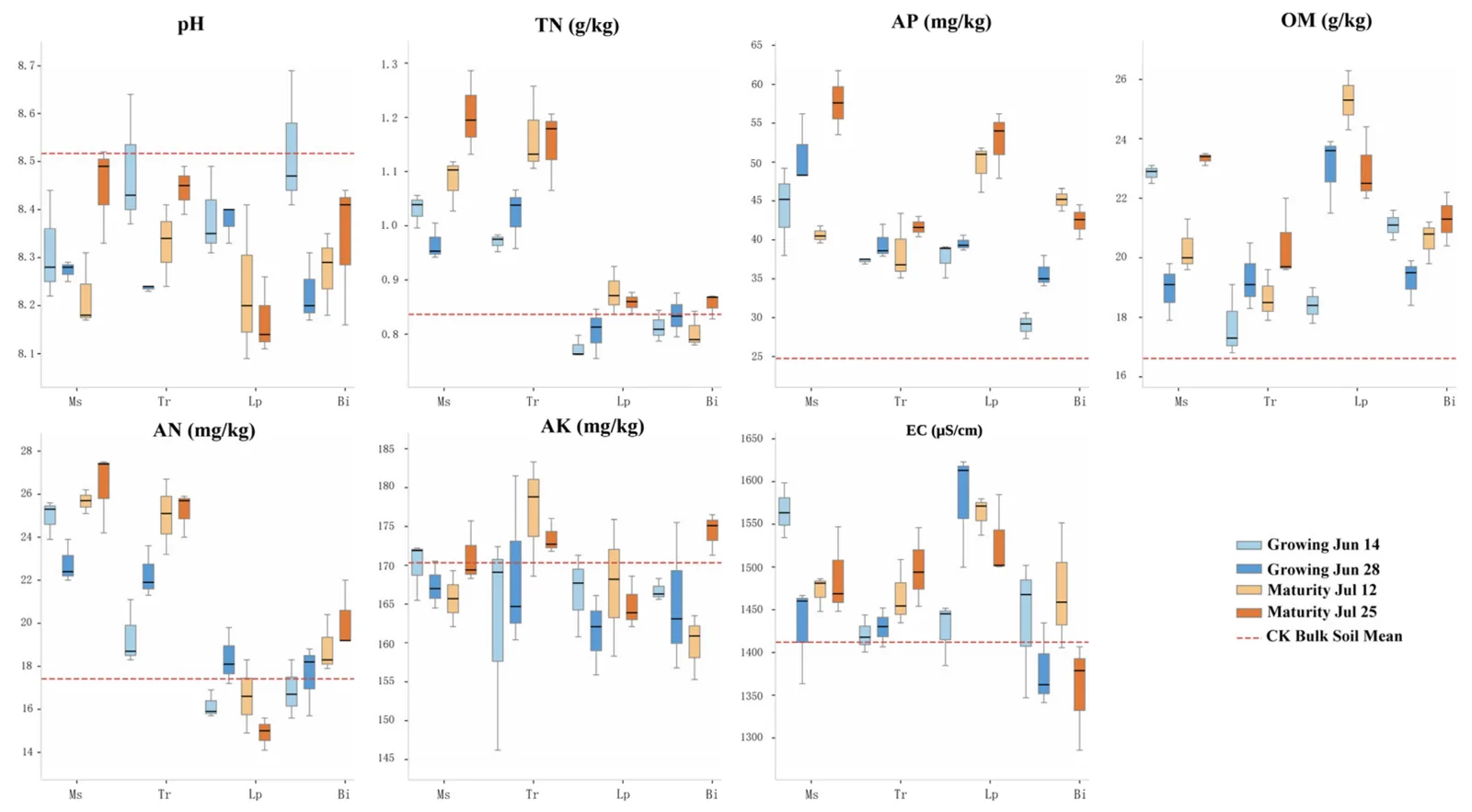
Box plots of seven physicochemical indices in rhizosphere soil across species and batches (*n* = 3 per box; the red dashed line indicates the across-batch overall mean of CK non-rhizosphere soil). Batch labels correspond to the BBCH-coded sampling dates defined in Section 4.1. Boxes show the median and interquartile range, with whiskers extending to 1.5 times the interquartile range; the standardized matrix of these data (together with Table 1) was used for the PCA and PERMANOVA.

**Figure 3:**
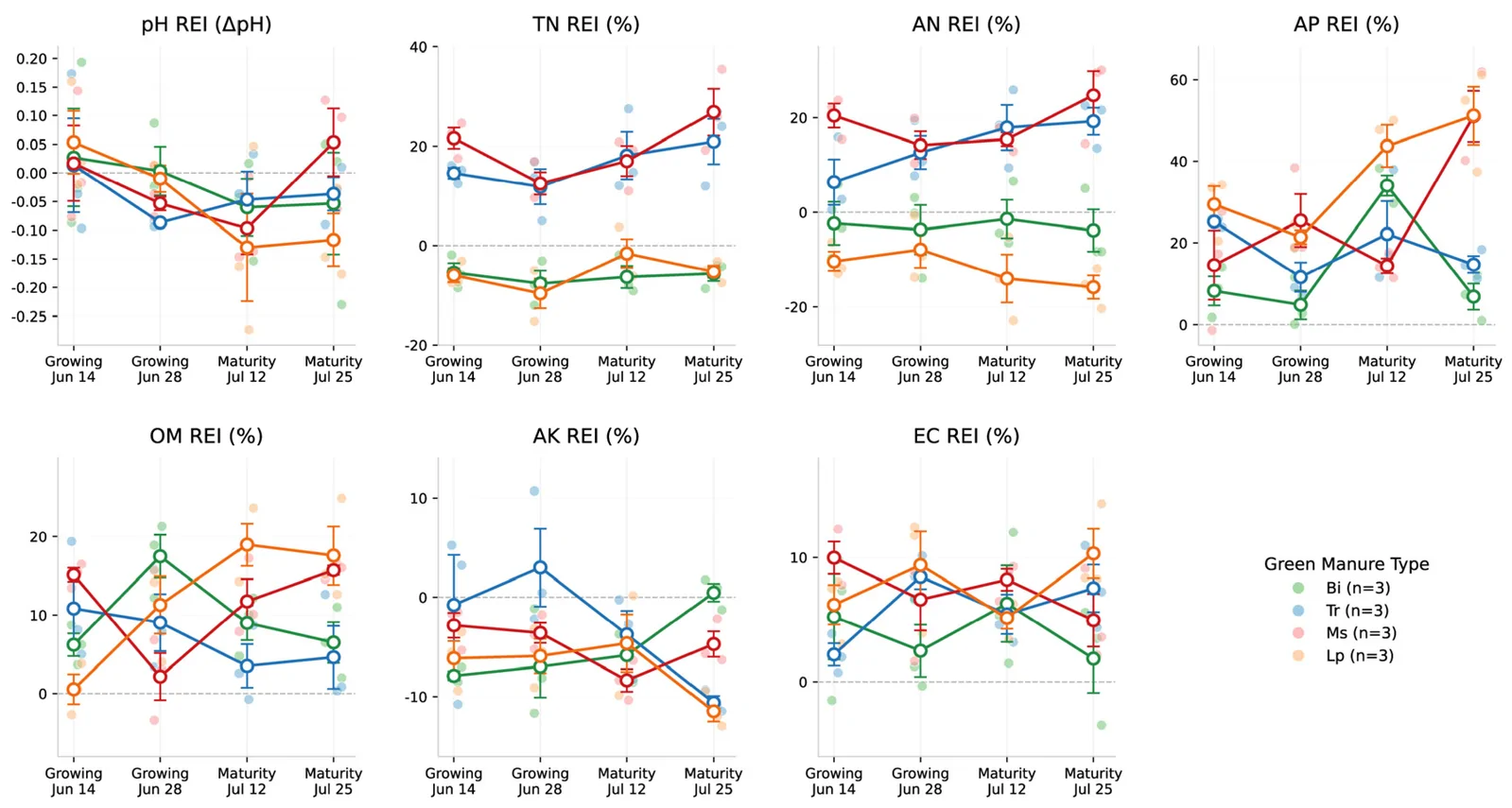
Temporal dynamics of rhizosphere effect index (REI) for each soil property (individual data points, *n* = 3). Batch labels correspond to the BBCH-coded sampling dates defined in Section 4.1. REI = (rhizosphere-non-rhizosphere)/non-rhizosphere × 100% (except pH, shown as absolute difference). Each point represents one biological replicate, and the REI was calculated sample-wise against the inter-row non-rhizosphere soil of the same species and batch; the REI matrix underlying this figure is the direct input of the Mantel, partial Mantel, and cross-stage covariation analyses. Colored lines/points represent different green manure species (Bi, Tr, Ms, Lp), and the horizontal dashed line indicates zero (0).

To make the statistical pathway fully traceable, the correspondence between figures and analyses is summarized here. Each figure corresponds to a specific analysis, but all statistics were computed from the underlying data matrices, not from the figures. Figure 1 displays the class-level chemotype composition on which the class-level two-way analysis of variance (species × stage) was computed (48 samples: 4 species × 4 stages × 3 replicates); it is descriptive, and no inferential statistic was derived from the plotted values. Figure 2 displays the rhizosphere soil data from which the standardized soil property matrix used for the PCA and PERMANOVA (n = 48) was built. Figure 3 displays the sample-wise REI values constituting the REI matrix used for the Mantel, partial Mantel, and cross-stage covariation analyses. The figures visualize, but are not the input of, the statistical analyses.

### 2.1. Species-Specific Chemotypes and Their Temporal Variation

A total of 112 root exudate compounds were identified across the four green manures, of which 84 were detected in *Ms*, 81 in *Lp*, 74 in *Bi* and 74 in *Tr*, and 65 were shared by all four species. *Ms* had 15 unique compounds, including pyrogallol, leucine and several sugars; *Lp* had 13, including oxalic acid, dihydrocaffeic acid, putrescine and erythritol; *Bi* had 8, including 2-butanol, hydroquinone, arginine and linoleic acid; and *Tr* had 7, including methyl myricetin, sitosterol and stigmasterol.

Despite substantial overlap in detected compounds, analysis of exudate classes revealed stable and distinct chemotypes. Chemotype naming was based on the mean of the seven classes across the four stages, and fluctuation at a single time point did not alter species classification. The chemotype in this study is a statistical classification based on the relative abundances of compound classes and describes only the compositional characteristics of the exudation profile; it does not represent a mechanistic functional classification at the level of metabolic pathways. *Ms* was a nitrogen-carbon-enriched type: amino acids and nitrogen-containing compounds had the highest relative abundance among the four species (mean 28.95%), together with the highest output of sugars and sugar alcohols (17.73%) and relatively high phenolics (20.02%); its advantage lay in carbohydrate diversity (16 compounds, including several unique sugars) rather than in the abundance of a single sugar such as glucose. *Lp* was an acid-phenolic-dominant type: small polar molecules (26.48%) and phenolics (22.63%) together accounted for 49.12%, with oxalic acid (batch means 11.32–19.41%) and dihydrocaffeic acid (9.57–13.04%) as unique markers. *Tr* was a dual-high nitrogen-acid type: amino acids (27.43%) and small polar molecules (20.42%) were both high; 3-hydroxyflavone reached 7.80% in the first maturation batch, the highest among the four species, and tryptophan increased from 6.48% in the growth stage to 10.91% at maturation. *Bi* was a multi-class balanced type: amino acids (25.15%), small polar molecules (21.54%), phenolics (18.58%), and long-chain aliphatic compounds (17.46%) were evenly distributed, and pyruvic acid increased from 5.07% in the growth stage to 9.63% at maturation.

Temporal dynamics were strongly species-dependent. *Bi* showed the strongest temporal restructuring: sugars decreased from 19.90% to 8.96%, small polar molecules increased markedly from 14.04% to 29.02%, and phenolics decreased from 20.71% to 15.45%, a pattern consistent with a shift from carbohydrate-dominated to organic acid-dominated exudation. In *Tr*, amino acids increased from 23.28% to 30.52% while sugars decreased from 16.64% to 10.03%. In *Lp*, small polar molecules (28.46% to 29.60%) and phenolics (23.80% to 22.04%) remained high, with only sugars increasing from 9.21% to 13.64%. *Ms* showed the smallest class-level fluctuation (amino acids from 29.09% to 26.73%; sugars from 20.57% to 16.76%), making it the only species with a temporally stable chemotype across all four stages.

### 2.2. Divergence in Rhizosphere Soil Properties and Species-Specific Rhizosphere Effect Indices

The seven rhizosphere soil indices diverged among species and showed consistent legume–grass differences (Figure 2). Here and throughout the Results section, stage and batch labels refer to the four sampling dates, with species-specific BBCH codes as defined in Section 4.1. Across all species, rhizosphere pH of the four green manures was lower than that of the clean-tilled control (8.52), with the smallest decrease in *Tr* (to 8.37) and the largest in *Lp* (to 8.29). Nitrogen indices differed clearly between legumes and grasses [4,25]. Rhizosphere TN and AN of *Ms* and *Tr* were higher than the control in all batches. For the two grasses, TN of *Lp* and *Bi* was generally slightly lower than the control, although *Lp* was slightly higher in the two maturation batches and *Bi* in the first growth and second maturation batches. The AN pattern differed between the grasses: *Bi* was slightly higher than the control in all batches (16.87–20.13 vs. 16.23–18.43 mg kg^−1^; overall means 18.36 vs. 17.42 mg kg^−1^), whereas *Lp* remained generally low (overall mean 16.51 mg kg^−1^) and exceeded the control only in the second growth batch (18.37 vs. 17.33 mg kg^−1^). AP and OM showed similar patterns of divergence: *Ms* had the highest absolute AP (48.33 mg kg^−1^), followed closely by *Lp* (44.89 mg kg^−1^), both clearly higher than the control; OM showed a similar pattern, with *Lp* (22.42 g kg^−1^) and *Ms* (21.35 g kg^−1^) higher than the control (16.62 g kg^−1^). In contrast, AK tended to decrease in the rhizosphere of all four species. Batch-to-batch scatter in some indices, such as the dispersion of *Tr* TN in the second growth batch, reflects the inherent spatial heterogeneity of the rhizosphere microdomain [21,26]. The absolute values of all seven indices for the CK, inter-row non-rhizosphere (background), and rhizosphere soils (means ± SD, *n* = 12 per treatment) are summarized in Table 1.

**Figure 4:**
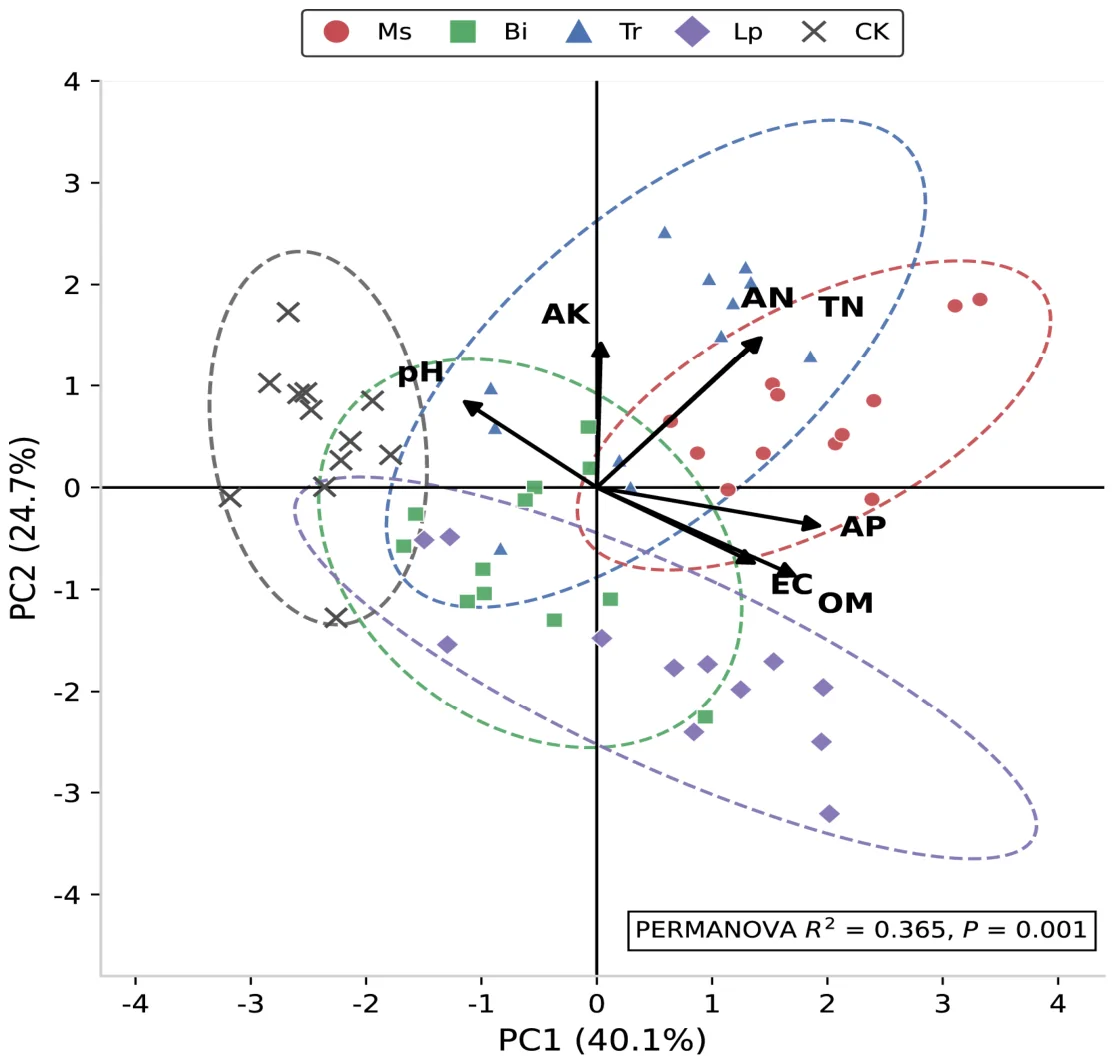
PCA ordination of rhizosphere soil properties, with CK non-rhizosphere soil as a reference. Arrows indicate variable loadings. PERMANOVA R^2^ = 0.365, *p* = 0.001. Dashed ellipses represent 95% confidence ellipses for each treatment group.

Relative to the CK baseline (pH 8.52, TN 0.84 g kg^−1^, AN 17.4 mg kg^−1^, AP 24.8 mg kg^−1^ and OM 16.6 g kg^−1^), Table 1 reveals clear species-specific improvement patterns. The legumes showed the strongest nitrogen enrichment: rhizosphere TN of *Ms* and *Tr* both reached 1.07 g kg^−1^, and AN rose to 24.9 and 23.0 mg kg^−1^, respectively, whereas rhizosphere TN and AN of the two grasses (*Bi*, *Lp*) were lower than in their respective background soils. For the increase in extractable P, *Lp* showed the largest gain relative to its background (+37.0%); absolute rhizosphere AP peaked in *Ms* (48.3 mg kg^−1^) and *Lp* (44.9 mg kg^−1^), and OM accumulation was highest in *Lp* (22.4 g kg^−1^). Rhizosphere pH decreased in all species (8.29–8.37 versus 8.52 in CK), with the smallest total decrease in *Tr* (0.15 units relative to CK). Rhizosphere AK of all four species (165.1–170.5 mg kg^−1^) was consistently lower than in their respective background soils (175.5–177.7 mg kg^−1^; CK 170.3 mg kg^−1^). These absolute values directly underpin the REI calculations and the PCA ordination in Figure 2, Figure 3 and Figure 4.

These absolute values describe the final state of the rhizosphere interface, but background differences in inter-row non-rhizosphere soil may mask the true modification capacity of each species. The REI, calculated against the inter-row non-rhizosphere soil of the same species and batch, removes background noise and focuses on the specific rhizosphere effect. On the REI basis, functional divergence among species was clearer (Figure 3). *Lp* showed the strongest increase in extractable P: rhizosphere AP increased by 37.0% relative to inter-row non-rhizosphere soil, OM by 12.4% and EC by 7.7%, while pH decreased by 0.051 units. This phosphorus gain was accompanied by marked nitrogen consumption, with rhizosphere TN and AN decreasing by 5.6% and 11.9%, and AK also decreasing by 7.1%, an asymmetric pattern of extractable P increase at the cost of nitrogen. *Ms* was the most balanced: rhizosphere nitrogen accumulation was the best among the species, with TN and AN increasing by 19.6% and 18.7%, while AP and OM also increased (by 14.5–51.0% and 2.2–15.7%, respectively), and the pH decrease was only 0.020 units, a synergistic pattern of concurrent nitrogen and phosphorus gains. The outstanding advantage of *Tr* was pH buffering: its inter-row non-rhizosphere soil pH was only 0.11 units lower than the control, and the rhizosphere decreased by a further 0.039 units, giving the smallest total plough layer decrease among the four species, with TN, AN and AP also increasing in a balanced manner. *Bi* was intermediate in most dimensions: AP and OM increased by 13.4% and 9.4%, but TN, AN and AK all declined, placing it between the legumes and *Lp* in overall rhizosphere impact. Rhizosphere AK of all four species was lower than the corresponding inter-row non-rhizosphere level, with decreases of 3.4% to 7.1%, confirming that potassium depletion is a common feature of green manure rhizospheres.

The rhizosphere effect did not increase monotonically over time; each index had its own fluctuation pattern. In *Lp*, AP-REI first fell from +29.4% to +21.4% and reached +51.1% in the second maturation batch, slightly higher than the +51.0% peak of Ms; however, based on the overall batch mean, Lp showed the strongest increase in extractable P (mean +37.0%, see Abstract). AP-REI of Ms rose from +14.5% to +51.0% with oscillations, whereas OM-REI fluctuated between +2.2% and +15.7% without a stable plateau. TN-REI of *Ms* rose from +21.6% to +26.9% at maturation. Overall, the temporal volatility of REI differed among species, echoing the inter-specific differences in chemotype stability observed in Section 2.1.

A three-way comparison of the control, inter-row non-rhizosphere soil, and rhizosphere soil allowed the effects of green manures on the plough layer to be summarized as two patterns. The first was a consistent effect: the decrease in pH and the increases in extractable P and OM occurred in the same direction in rhizosphere and inter-row non-rhizosphere soils, forming a continuous gradient from the control to inter-row non-rhizosphere soil and then to rhizosphere soil. Specifically, the inter-row non-rhizosphere pH of the four species decreased by 0.11 to 0.18 units relative to the control, with a further decrease of 0.02 to 0.05 units in the rhizosphere; inter-row non-rhizosphere AP increased by 32.4% to 54.4% relative to the control, with a further increase of 13.4% to 37.0% in the rhizosphere; and inter-row non-rhizosphere OM increased by 7.2% to 20.0% relative to the control, with a further increase of 6.8% to 12.4% in the rhizosphere. These improvements may be driven jointly by physical effects [27], such as root penetration and shading with moisture conservation, and by chemical effects of exudates. The second was a divergent effect: inter-row non-rhizosphere AK of the four species increased by 3.0% to 4.3% relative to the control, whereas rhizosphere soil was 3.4% to 7.1% lower than the inter-row non-rhizosphere soil of the same species, so that potassium depletion was strictly confined to the rhizosphere interface. The two grasses increased TN and AN by 5.0% to 8.5% in inter-row non-rhizosphere soil relative to the control, whereas their rhizosphere soils were 2.8% to 11.9% lower than their own inter-row non-rhizosphere soils, again confining nitrogen depletion to the rhizosphere. Legumes showed the opposite pattern: Their nitrogen surplus spilled over from the rhizosphere to the inter-row non-rhizosphere soil, and the total nitrogen (TN) content in this soil was 7.1–10.5% higher than that of the control.

### 2.3. Systematic Improvement of Rhizosphere Soil Properties: Evidence from Multivariate Ordination

The PCA ordination resolved the divergence pattern of the four green manure rhizosphere soils in two-dimensional space (Figure 4). PC1 (40.1% of variance) was characterized by high positive loadings of AP (+0.88), OM (+0.78), and EC (+0.62), and a negative loading of pH (−0.52), forming a phosphorus–OM–acidification gradient; *Lp* plots lay farthest to the right of this axis, *Ms* and *Tr* were positioned intermediately and shifted to the right, *Bi* was close to the origin, and the CK cluster was concentrated in the left quadrant. PC2 (24.7% of variance) was defined by TN (+0.67), AN (+0.67), and AK (+0.65) as a nitrogen–potassium gradient; *Ms* and *Tr* scored positively and clustered together along this axis, whereas the two grasses were distributed below or near the origin, forming a clear legume–grass boundary along PC2. The four species radiated outward from the CK centroid: *Ms* occupied the upper-right quadrant, *Lp* shifted horizontally to the right along PC1, *Tr* was intermediate with a positive PC2 score, and *Bi* showed the smallest displacement. PERMANOVA based on the 48 replicate samples [28] (4 species × 4 batches × 3 replicates, *n* = 48) confirmed significant clustering of the four green manures in PCA space (F = 8.44, R^2^ = 0.365, *p* = 0.001). Species identity explained 36.5% of the variation, indicating that the overall variation of the seven rhizosphere soil indices was strongly driven by species identity, and that the improvement effects of different green manures diverged systematically along the orthogonal phosphorus–OM and nitrogen–potassium pathways.

### 2.4. Hierarchical Associations Between Exudate Chemotypes and Soil Properties

**Figure 5:**
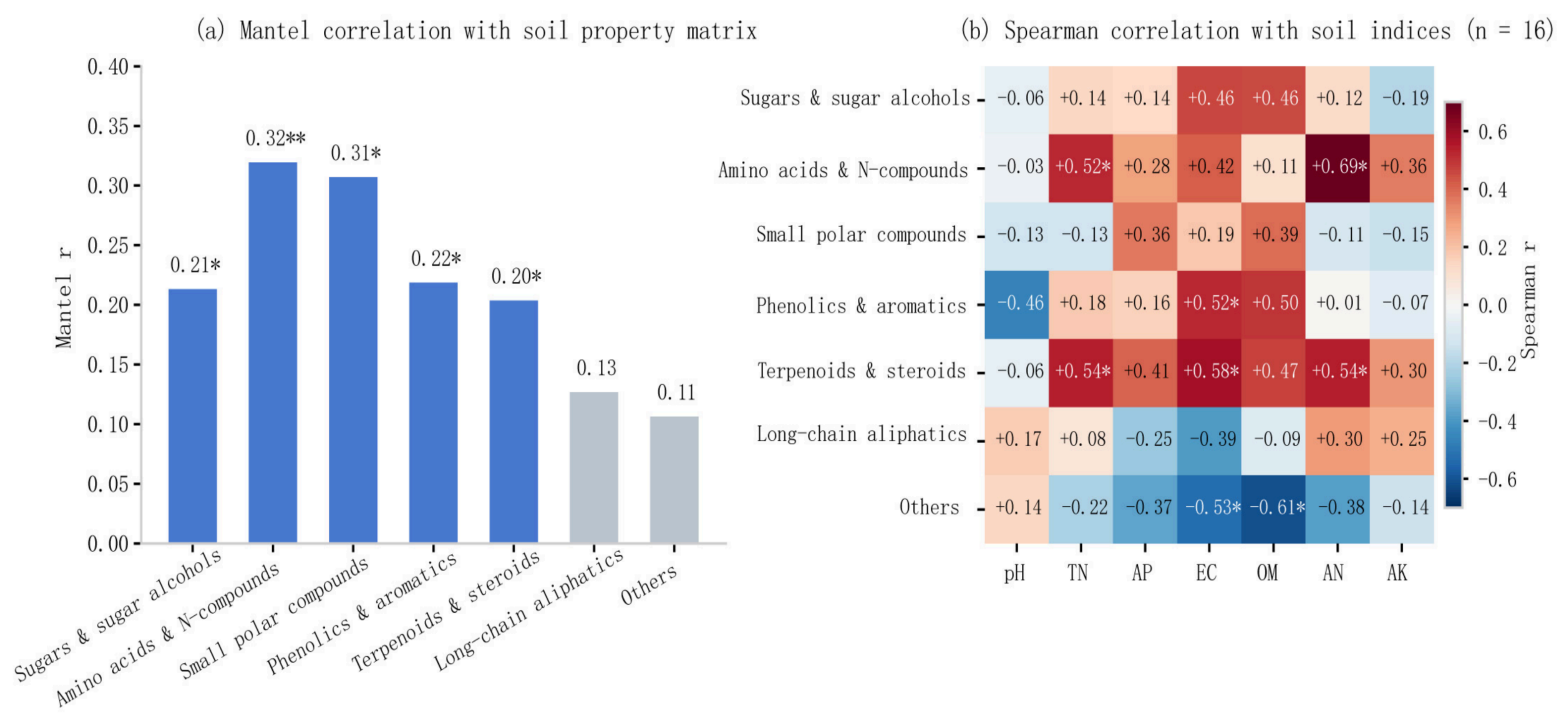
Associations between exudate compound classes and rhizosphere soil physicochemical properties based on CLR-corrected compositional data. (**a**) Mantel coupling strength between the within-class subcomposition of each exudate class (within-class peak areas reclosed followed by CLR transformation; Euclidean distance of CLR coordinates) and the standardized soil property matrix (Euclidean distance) based on species × stage centroids (*n* = 16; 999 permutations). * *p* < 0.05, ** *p* < 0.01; r values are shown above the bars. (**b**) Spearman correlation coefficients between class-level CLR coordinates (CLR applied to the seven class relative abundances after zero replacement) and individual soil indices (*n* = 16). * *p* < 0.05 before multiple-testing correction; none of the 49 class–index pairs remained significant after Benjamini–Hochberg correction (minimum q = 0.157); values are shown for direction only. For the class others, *n* = 15 because the *Lolium perenne* centroid at the first sampling date (14 June) was excluded, as neither constituent compound was detected.

The Mantel test, based on 16 species [29]–stage centroids (4 species × 4 stages, 999 permutations) and Aitchison distances of CLR-corrected exudate composition, showed that the overall exudate chemotype was significantly coupled to the soil property matrix (*r* = 0.309, *p* = 0.002) [30,31]. At the class level, amino acids and nitrogen-containing compounds (*r* = 0.32, *p* < 0.01), small polar molecules (*r*= 0.31, *p* < 0.05), phenolics and aromatics (*r* = 0.22, *p* < 0.05), sugars and sugar alcohols (*r* = 0.21, *p* < 0.05) and terpenoids, steroids and lipophilic signal molecules (*r* = 0.20, *p* < 0.05) were all significantly coupled to the soil property matrix, whereas long-chain aliphatic compounds and the class ‘others’ were not significant (Figure 5a). Class–index Spearman correlations (Figure 5b) showed that 8 of 49 pairs had uncorrected *p* < 0.05 (*p* = 0.003–0.037), but none remained significant after Benjamini–Hochberg [32] correction (minimum *q* = 0.157). In direction, amino acids and nitrogen-containing compounds showed the highest uncorrected positive correlation with AN (*r* = +0.69, uncorrected *p* = 0.003) and a moderate positive correlation with TN (*r* = +0.52), ranking first among all class–nitrogen index pairs (7 classes × 2 N indices) and consistent in direction with the strongest class–level matrix coupling in Figure 5a. Terpenoids, steroids, and lipophilic signal molecules were positively correlated with TN (*r* = +0.54), AN (*r* = +0.54), and EC (*r* = +0.58), and phenolics and aromatics were moderately positively correlated with EC (*r* = +0.52). The strongest negative correlations were those of the class ‘others’ with OM (*r* = −0.61) and EC (*r* = −0.53). Coupling between classes and soil properties was expressed mainly at the level of the multi-index matrix (Figure 5a); coefficients for single indices indicate only the direction and relative strength of association. The class ‘others’ contained only two trace phthalate compounds (mean relative abundances below 0.01%), and its nominally significant negative correlations before multiple-testing correction may reflect trace-level noise and should not be interpreted mechanistically.

**Figure 6:**
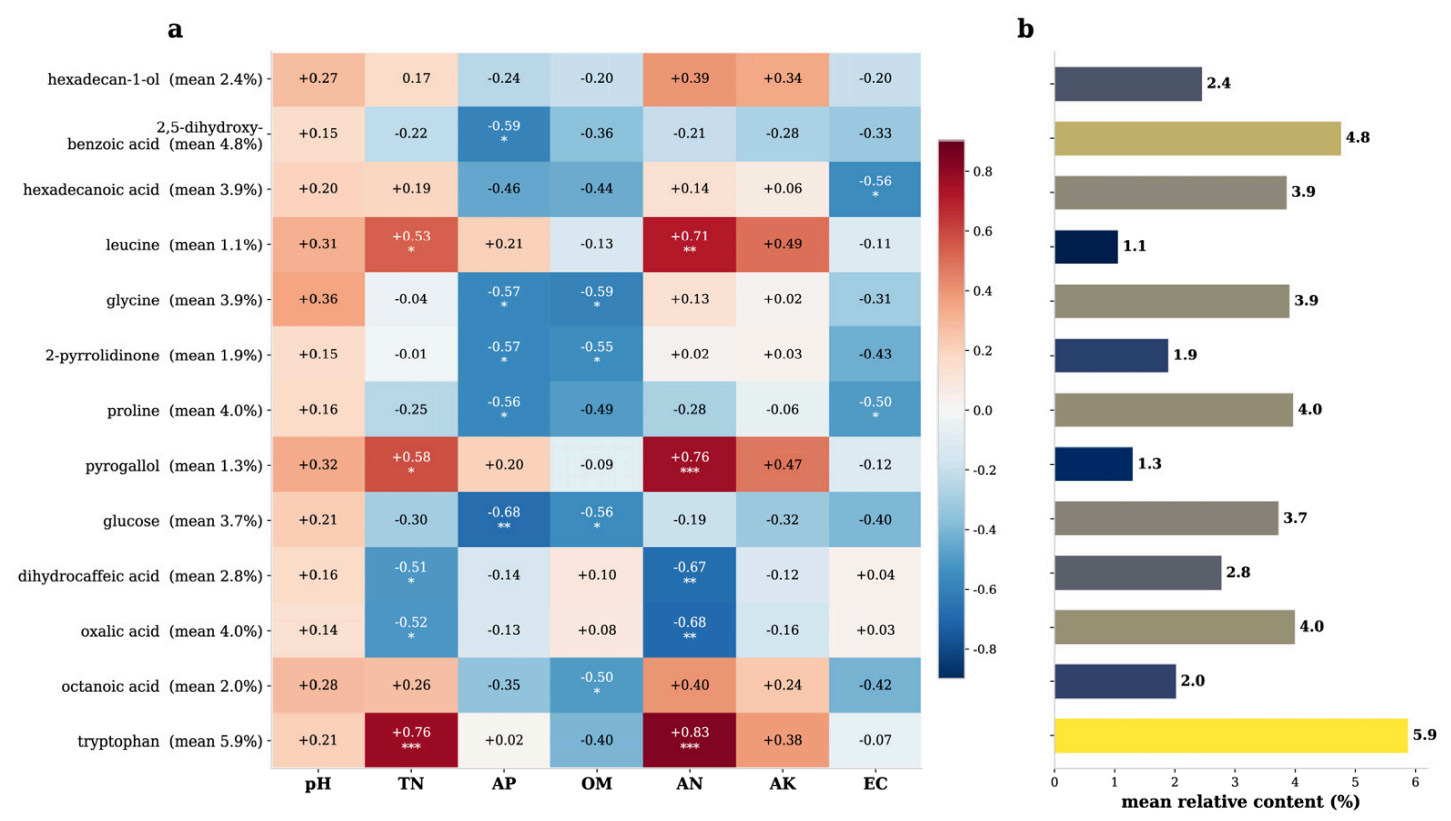
Spearman correlation heatmap between high-content representative compounds and rhizosphere soil physicochemical indices (**a**), based on CLR-corrected compositions at species × stage centroids (*n* = 16; * *p* < 0.05, ** *p* < 0.01, *** *p* < 0.001 before multiple-testing correction; none remained significant after Benjamini-Hochberg correction across all 784 tests), and mean relative content of each compound (**b**).

To test whether class-level effects were driven by overall class flux or by a few high-abundance single molecules, representative molecules were screened by a dual criterion: (i) high content (centroid mean ≥ 1.5%, or reaching ≥ 5% in any single batch) and (ii) strong association (|r| ≥ 0.5 and uncorrected *p* < 0.05). Among 13 candidate molecules, tryptophan (amino acid class) showed the highest uncorrected positive correlations [33,34] with AN (*r* = +0.83, *p* < 0.001) and TN (*r* = +0.76, *p* < 0.001), consistent with the class-level trend of amino acids; pyrogallol (phenolics, *r* = +0.76, *p* < 0.01) and leucine (amino acids, *r* = +0.71, *p* < 0.01) were also strongly positively correlated with AN. In contrast, the negative correlation of glucose (sugars) with AP (*r* = −0.68, uncorrected *p* < 0.01) was inconsistent with the non-significant overall pattern of the sugar class. None of the 784 compound–index tests remained significant after BH-FDR correction (minimum *q* = 0.056). All single-molecule correlation values reported here are therefore uncorrected results and are used for exploratory reference only. These effect sizes, such as the uncorrected correlations of +0.76 to +0.83 between tryptophan and rhizosphere nitrogen, can be used to generate hypotheses for follow-up studies but cannot serve as confirmatory evidence. Replicate-level analysis (*n* = 48) showed that the correlations of tryptophan with TN (r = +0.80, *p* < 0.001) and AN (r = +0.76, *p* < 0.001) persisted at the replicate scale (Figure S1) and also held within species (Tr, *n* = 12: r = +0.78 with TN and r = +0.59 with AN, both uncorrected *p* < 0.05). Because replicates within a species × stage combination are not fully independent observations, the replicate-level result was used as supportive evidence, with the centroid-level analysis (Figure 6a) remaining the primary inference. Tryptophan was a high-abundance amino acid in all four species (overall mean 5.87%), with legume output (*Ms* 9.90–10.38%; *Tr* 6.48–10.91%) significantly higher than grass output (1.68–2.84%).

Among the remaining candidate compounds, leucine was moderately positively correlated with TN (*r* = +0.53) and pyrogallol with TN (*r* = +0.58) (Figure 6); leucine and pyrogallol were highly expressed only in *Ms*. In the negative direction, several compounds showed uncorrected correlations with soil indices: oxalic acid and dihydrocaffeic acid with AN (*r* = −0.68 and −0.67, respectively; Figure S2c,d), glucose with AP and OM (*r* = −0.68 and −0.56), glycine with AP and OM (*r* = −0.57 and −0.59), 2,5-dihydroxybenzoic acid with AP (*r* = −0.59), and proline with EC (*r* = −0.50; Figure S2f). Although the batch mean of oxalic acid in *Lp* was as high as 11.32–19.41%, no significant dose relationship with AP was detected at the within-species replicate level (*n* = 12, *p* > 0.05; Figure S2).

### 2.5. Temporal Covariation and Family-Level Background of the Chemotype–Soil Coupling

**Figure 7:**
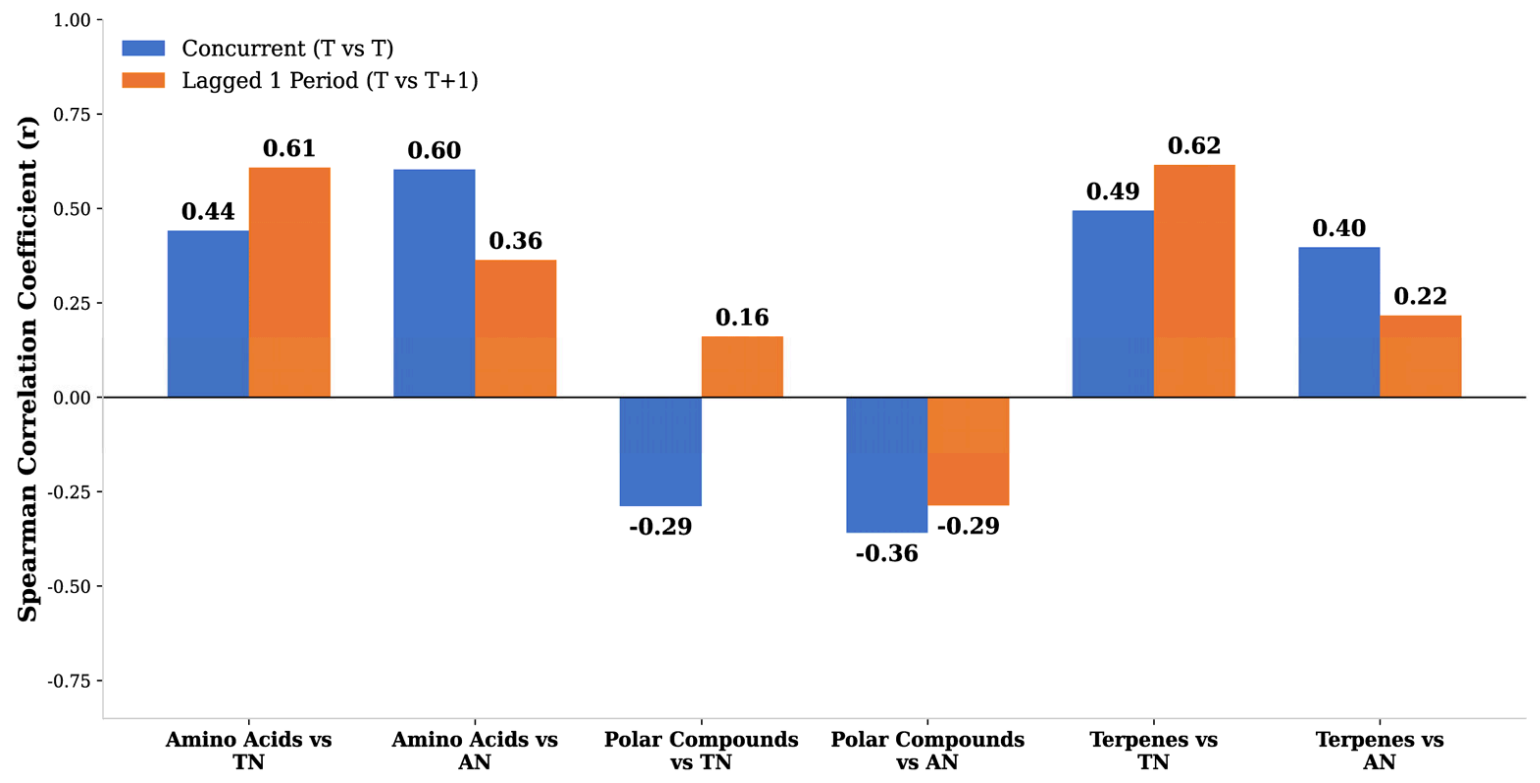
Concurrent and cross-stage Spearman correlation coefficients between exudate classes and soil REI based on CLR-corrected compositions. Bar chart comparing the concurrent (stage T versus stage T, *n* = 16) and cross-stage (stage T versus stage T + 1, *n* = 12) Spearman correlation coefficients for six key class-REI pairs; r values are shown above the bars. None of the 12 pairs remained significant after Benjamini-Hochberg correction (minimum q = 0.14).

The sampling interval of approximately 13–14 days in this study is far longer than the 2–8 h scale of rhizosphere solute turnover reported in the literature [35]; the cross-stage correlations here can therefore reflect only short-term co-fluctuation across sampling stages, which we term cross-stage covariation analysis. Spearman cross-correlations between CLR-corrected exudate classes at stage T and soil REI at stage T + 1 (Figure 7) showed that, overall, the covariation signals were weak after CLR correction and none remained significant after multiple-testing correction. In concurrent correlations, the positive covariation of amino acids with AN-REI reached uncorrected *p* < 0.05 (r = +0.60), and the covariation of amino acids and terpenoids-steroids with TN-REI was marginally significant (r = +0.44 and +0.49, *p* < 0.10). Within the first cross-stage window, the positive covariation of amino acids and terpenoids-steroids with TN-REI persisted (r = +0.61 and +0.62, uncorrected *p* < 0.05), whereas the covariation with AN-REI weakened, with amino acids falling from +0.60 to +0.36. Small polar molecules showed only weak negative covariation with AN-REI, significant in neither case (concurrent r = −0.36; cross-stage r = −0.29; *p* > 0.10). None of the 12 pairs remained significant after Benjamini–Hochberg correction (minimum q = 0.14).

**Figure 8:**
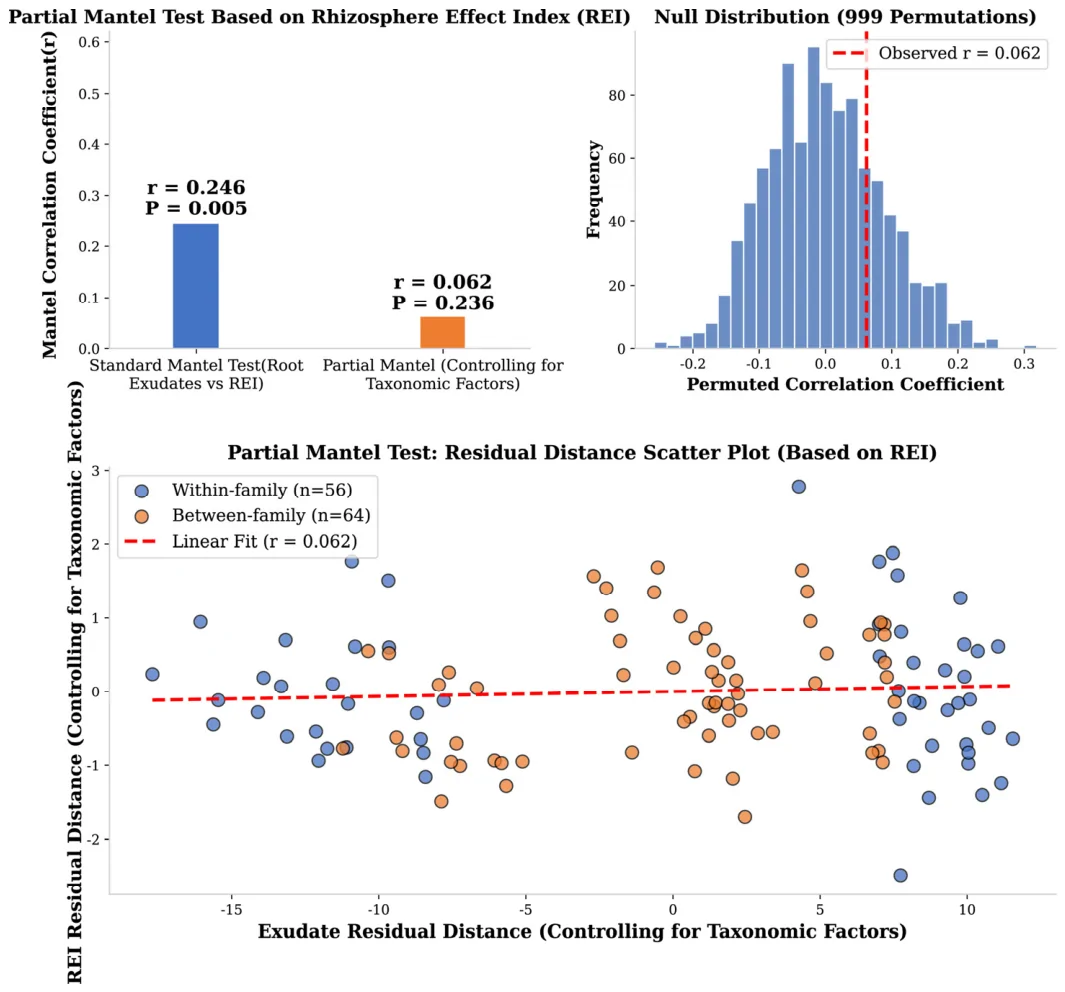
Partial Mantel test controlling for family-level background and residual validation based on CLR-corrected compositions. (**a**) Standard Mantel test (exudates versus REI, r = 0.246, *p* = 0.005), partial Mantel test controlling for family (r = 0.062, *p* = 0.236) and the 999-permutation null distribution; the coupling is largely shaped by family-level background. (**b**) Residual distance scatterplot after controlling for family. Blue points are within-family sample pairs (*n* = 56), orange points are between-family sample pairs (*n* = 64), and the red dashed line is the fitted regression line (partial Mantel *r* = 0.062). The comparison is based on two species per family and reflects the legume-grass contrast among the four studied species rather than a general phylogenetic pattern.

The partial Mantel test showed that the standard Mantel correlation (exudates versus REI, based on the z-standardized REI matrix rather than the raw soil property matrix used in Section 2.4) was r = 0.246 (*p* = 0.005) and fell to r = 0.062 (*p* = 0.236) after controlling for family, indicating that the dominant component of this coupling cannot be separated from family-level background (Figure 8). This r value is therefore not directly comparable to the r = 0.309 reported in Section 2.4, as the two tests use different response matrices. This analysis is based on only two species per family and therefore reflects the legume–grass contrast among the four species studied here, rather than a general phylogenetic pattern. Residuals of within-family (*n* = 56) and between-family (*n* = 64) sample pairs were intermingled around the regression line with no obvious stratification. Attributing the coupling to family-level background does not diminish the operational value of the chemotype for green manure selection, because field configuration is inherently a species-level decision, and a chemotype index measurable in a single GC-MS batch remains a practical bridge between species identity and soil function, whatever its evolutionary origin (see Section 2.6 for the comprehensive evaluation and Section 3.1 for further discussion).

### 2.6. Target-Oriented Evaluation of Green Manures Anchored to the Nitrogen Function Axis

The evaluation window covered the two maturation stage batches (12 and 25 July; BBCH stage codes as defined in Section 4.1), for which 21 amino acids and nitrogen-containing compounds were semi-quantified with norvaline as the internal standard. The maturation stage was chosen because exudate output at this stage is directly related to nutrient release after green manure mowing or incorporation. Because soil AN at the experimental site was low (CK 16.23–18.43 mg kg^−1^) and, as shown in Section 2.4, the class of amino acids and nitrogen-containing compounds showed the strongest matrix-level coupling with soil properties (Mantel *r* = 0.32, *p* < 0.01), the evaluation was oriented towards nitrogen function.

This evaluation is target-oriented: index selection was based on class-level matrix coupling strength (the amino acid class) rather than on single-molecule statistical significance. Although tryptophan showed a strong exploratory correlation with nitrogen indices in Section 2.4 (uncorrected *r* = +0.83), it did not reach the significance threshold after multiple-testing correction and therefore has no independent causal indicative meaning. In the evaluation, tryptophan was included only as the most variable measurable component within the amino acid class for sensitivity analysis; this does not imply any functional driving effect. Importantly, class abundance reflects the input flux of nitrogen-containing compounds from roots, rather than the net rhizosphere nitrogen balance—the latter is influenced by microbial consumption, plant reabsorption, and leaching, and is better captured by the soil TN and AN data presented in Section 2.2.

To prevent a single unverified molecule from unduly influencing the conclusion, the two-index scenario excluding tryptophan (class abundance plus Tstab) was used as the core evaluation framework; the three-index scenario including tryptophan was used only as a sensitivity analysis to test whether this molecule would change the final species order. Tstab measures the temporal inertia of class abundance within the maturation period and serves to correct for possible accidental upward or downward bias of the class abundance mean in a single sampling; it is not an independent abundance-type index for comparison. Its weight in the entropy method reflects the additional information of temporal reliability and does not directly represent nitrogen supply capacity.

Under the core framework, the entropy weights of class abundance and Tstab were 0.695 and 0.305, respectively. TOPSIS closeness ranked the species as *Ms* (0.9621) > *Bi* (0.2857) > *Tr* (0.2013) > *Lp* (0.0000), and gray relational grade ranked them as *Ms* (0.9335) > *Bi* (0.5650) > *Tr* (0.4610) > *Lp* (0.3333), with *Ms* firmly first.

**Figure 9:**
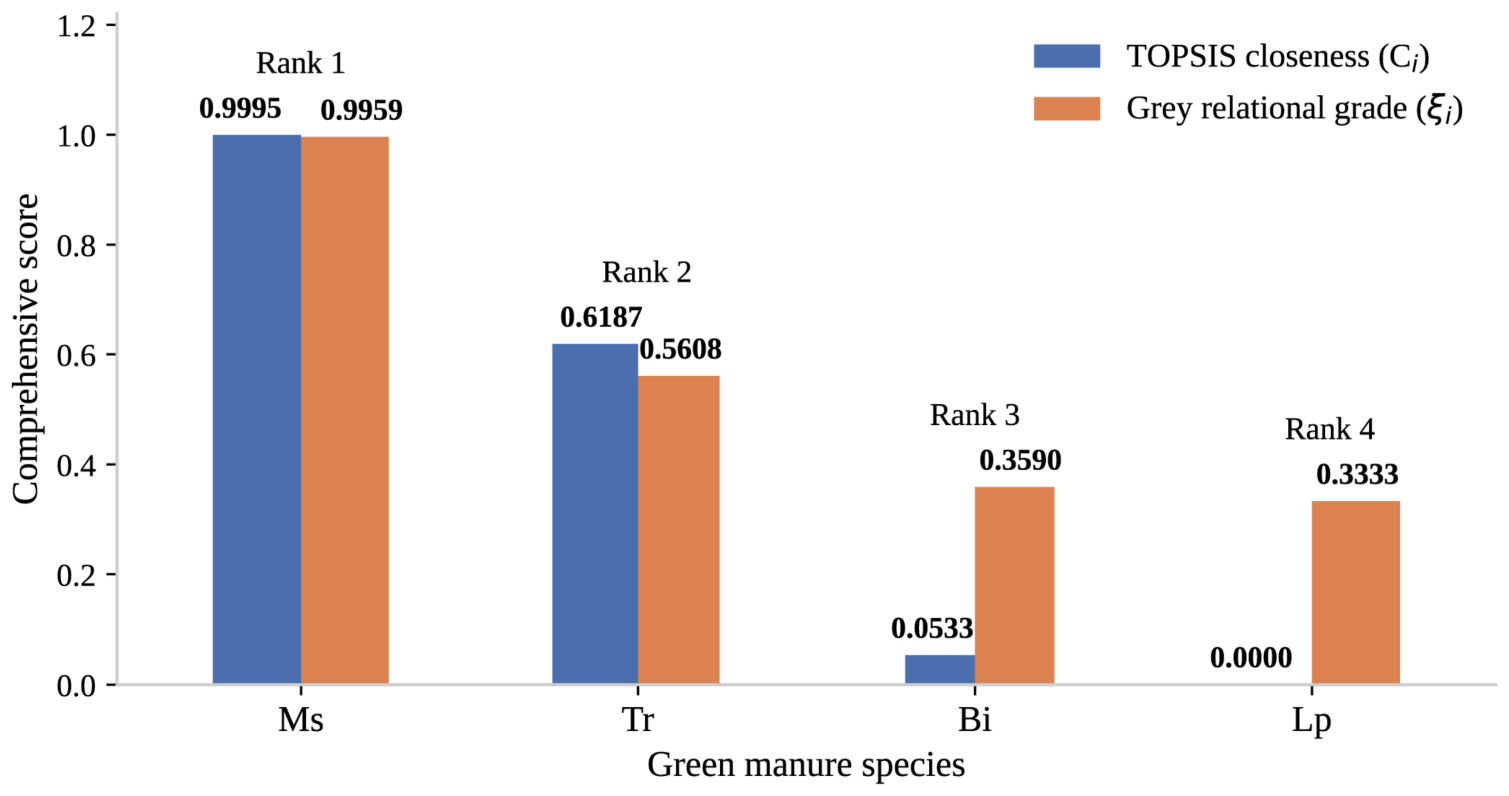
Comprehensive evaluation scores of the four green manures based on internal standard semi-quantification of amino acids and nitrogen-containing compounds at the maturation stage with entropy weighting (three-index sensitivity scenario). Blue bars indicate TOPSIS closeness C and orange bars indicate gray relational grade ξ, with scores labeled above the bars and overall ranks shown at the top.

In the three-index sensitivity analysis including tryptophan (Table 2, Figure 9; the core framework results are reported in the text), the entropy weights of class abundance, tryptophan, and Tstab were 0.043, 0.938, and 0.019, respectively. The extremely high weight of tryptophan arises from its numerical dispersion among species and reflects a mathematical property rather than ecological importance. Under this scenario, TOPSIS ranked the species as *Ms* (0.9995) > *Tr* (0.6187) > *Bi* (0.0533) > *Lp* (0.0000), and gray relational analysis ranked them as *Ms* (0.9959) > *Tr* (0.5608) > *Bi* (0.3590) > *Lp* (0.3333). Under the equal-weight sensitivity test of the three-index scenario, the TOPSIS order was unchanged, whereas *Bi* and *Tr* swapped their second positions in gray relational analysis.

Across all four scenarios—the two-index core framework, the three-index TOPSIS, the three-index GRA, and the equal-weight TOPSIS—*Ms* retained first place in all cases, showing that the recommendation does not depend on whether tryptophan is included, on weighting values, or on the evaluation algorithm. This ranking is mutually corroborated, along the nitrogen function dimension, by the measured rhizosphere TN-REI of *Ms* (+19.6%), the best among the four species in Section 2.2.

## 3. Discussion

### 3.1. Causal Boundaries and Hierarchical Mechanisms of the Chemotype–Soil Coupling

The 36.5% species effect from PERMANOVA and the Mantel coupling (*r* = 0.309) address different questions: the former measures among-species soil divergence; the latter tests whether chemotype captures variation parallel to soil properties. Exudate composition alone was significantly coupled to soil divergence, with amino acids showing the strongest class-level coupling. Controlling for family reduced coupling to *r* = 0.062, indicating the dominant component cannot be separated from family-level background. For green manure screening, this does not diminish the chemotype’s practical value: field configuration operates at the species level, and a chemotype index measurable in a single GC-*MS* batch is an operational bridge between species identity and soil function [22,23]. Future controlled experiments could test the causal efficacy of the chemotype signal.

Class-level Mantel coupling was dominated by amino acids (*r* = 0.32) and small polar molecules (*r* = 0.31). The partial Mantel test (*r* = 0.246 → 0.062 after controlling for family) suggests this reflects conserved metabolic differences between legumes and grasses rather than a universal small-molecule mechanism. Because only two species per family were included, this coupling pattern cannot be extrapolated to broader phylogenetic scales. Tryptophan showed the highest uncorrected single-molecule correlation with rhizosphere nitrogen (*r* = +0.83) but did not survive BH-FDR correction, and is statistically indistinguishable from family-level background. Tryptophan should therefore be positioned as an auxiliary indicator, not a driver of nitrogen accumulation. Assessment of nitrogen-cycle function should focus on the amino acid class as a whole.

It should also be stressed that detecting the seven compound classes itself says nothing about nutrient mobilization; each class maps onto specific soil chemical pathways whose net outcome must be verified by soil measurement. Small polar molecules and organic acids can promote ligand exchange at mineral surfaces and, through proton release and metal chelation, dissolve Ca-phosphates, although in calcareous soils much of the solubilized phosphorus may be re-adsorbed or re-precipitated; phenolics and aromatics participate in metal complexation and redox reactions and modulate pH buffering; amino acids and nitrogen-containing compounds enter microbial mineralization–immobilization turnover, so their rhizosphere signal reflects net nitrogen release rather than gross input; sugars and sugar alcohols fuel heterotrophic activity and the associated priming of organic matter; and long-chain aliphatic compounds contribute mainly to soil hydrophobicity and aggregate stabilization. The class–soil couplings reported here are therefore interpreted as associations with the net outcomes of these pathways, not as direct demonstrations of nutrient mobilization [18].

The dominance of family-level signal in the chemotype–soil coupling does not, however, render chemotype characterization redundant. First, the chemotype remains a valid high-dimensional descriptor of species-specific exudation strategies: even when family-level background accounts for most of the covariance, the residual within-family variation—such as the distinct temporal inertia of Ms versus Tr—still carries biologically meaningful information about functional stability and developmental plasticity. Second, the chemotype framework provides a compositional reference against which deviations can be detected; the fact that association strength collapses under family-level control does not mean that exudate profiles are uninformative, but rather that their predictive power for soil outcomes is largely inherited at the family level and should not be over-attributed to single compounds or species-specific idiosyncrasies. Third, in practical screening, chemotype classification can serve as a rapid, cost-effective proxy for broad functional groups (e.g., N-enriched legume type vs. acid-phenolic grass type), guiding initial selection before costly long-term field trials are undertaken. Thus, even under a strong family-structured background, chemotype characterization retains heuristic and screening value, provided its correlational nature and hierarchical position are clearly communicated.

A further limitation is the absence of microbiological evidence. At realistic rhizosphere concentrations, root exudates rarely modify soil chemistry directly; most of their effects are transduced by microorganisms [36]—phosphorus-solubilizing bacteria releasing phosphatases, nitrogen-cycling guilds driving transformations, and heterotrophs mineralizing organic matter. Our design observes only the two ends of the chemotype–soil property chain, leaving the microbial link a black box. Two considerations push this point further. First, because the Mantel coupling collapsed from *r* = 0.246 to 0.062 once family was controlled (Section 2.5), the coupling is likely carried to a large extent by microbial mediation rather than by the measured molecules themselves. Second, legume and grass rhizosphere microbiomes are themselves systematically differentiated, so the family-level divergence of chemotypes may partly mirror a phylogenetically structured rhizosphere microbiome; in other words, the chemotype may act as a proxy of the holobiont [37] rather than as the causal agent itself. This interpretation does not diminish the practical value of chemotypes in green manure screening, but it clearly delimits the boundary of mechanistic understanding. We therefore propose the following validation route: paired 16S/ITS amplicon and metagenomic sequencing of the same 1–4 mm microdomain; measurement of functional genes corresponding to the implicated processes (e.g., *phoD* for organic-phosphorus mineralization [38], *nifH* and *amoA* for nitrogen fixation and nitrification) together with extracellular enzyme activities; ^13^C-labeled exudates combined with DNA/RNA stable-isotope probing to identify the microbial taxa that actually consume the dominant compounds of each chemotype; and, ultimately, re-inoculation of synthetic communities into sterilized rhizosphere soil, which would convert the present associational chemotype–soil link into a testable mechanistic chain.

### 3.2. Two-Layer Structure and Divergence of Nutrient-Acquisition Strategies

The three-way comparison revealed two spatial patterns. pH, AP, and OM formed continuous gradients from CK to inter-row non-rhizosphere to rhizosphere soil; AK and grass nitrogen showed opposite differences. The former reflects combined physical and chemical effects; the latter reflects root uptake and root–microbe competition. Using REI alone would underestimate total plough layer improvement.

A further caveat concerns the spatial asymmetry behind the REI itself: its numerator comes from the 1–4 mm root sheath microdomain, whereas its denominator is the 0–20 cm inter-row background soil, and the two differ in both spatial volume and temporal integration. The REI is therefore an interface-scale intensity measure rather than a plough layer extensive quantity: it answers questions of direction and ranking, not how much an entire field has improved. Three consequences follow. First, absolute REI values cannot be linearly extrapolated to field-scale nutrient budgets—even the largest effect observed here (rhizosphere available P +37.0% in *Lp*) acts on a hot-spot soil mass orders of magnitude smaller than the background soil that forms the denominator, and the actual plough layer consequence depends on the hot-spot-to-background volume ratio and on how far the signal propagates outward, neither of which was measured. Second, the root sheath turns over within days, whereas the 0–20 cm background integrates the whole growing season, so part of the batch-to-batch REI fluctuation in Figure 3 may reflect this temporal mismatch rather than genuine biological switching. Third, the asymmetry in spatial granularity can even reverse the interpretation of depletion signals: rhizosphere AK of all four species was 3.4–7.1% lower than their respective backgrounds, yet inter-row AK of all species exceeded that of the clean-tilled control (175.5–177.7 versus 170.3 mg kg^−1^); in other words, the same species depletes potassium at the interface while enriching it at the plot scale, and the REI captures only the former. We therefore interpret plough layer improvement as the product of interface intensity, hot-spot extent and persistence, and we consider the parameterization of hot-spot geometry (root length density and root sheath thickness) combined with mass-balance models that scale the REI to the plough layer a priority for future work [39]. In addition, the 1–4 mm sampling layer is wider than the conventional definition of the rhizosphere, within which the steepest gradients of most parameters occur at 1–2 mm from the root surface [18]; the collected layer thus averages the rhizosphere proper with a transition zone, and the REI should be read as an interface-scale comparison rather than a strict rhizosphere measurement.

It should be clarified that the REI normalizes background differences but not uptake differences. Because the REI is calculated against the inter-row non-rhizosphere soil of the same species and batch, it removes inter-specific differences in background soil levels and focuses the comparison on the increment at the rhizosphere interface relative to each species’ own background. Nutrient uptake, however, acts mainly within the rhizosphere microdomain, whereas the inter-row background soil is distant from the roots and is only weakly affected by the uptake of that species; the REI therefore does not normalize uptake differences and may even retain or amplify their signal, because stronger uptake lowers rhizosphere nutrient concentrations relative to the background. The soil effects reported here can therefore not be attributed to root exudates alone, and separating exudation from uptake must rely on independent pattern evidence. Three such patterns indicate that nutrient removal by uptake cannot fully explain the observed soil differences. First, the spatial reversal described above: the same species depletes potassium at the interface while enriching it at the plot scale, which is difficult to reconcile with uptake removal alone. Second, the legume–grass contrast: if uptake removal dominated, the more N-demanding legumes should show stronger rhizosphere N depletion, yet rhizosphere TN and AN of *Ms* and *Tr* were higher than their own backgrounds (TN-REI of *Ms* reaching +19.6%; Section 2.2), a nitrogen surplus more consistent with biological N fixation input, rhizosphere N turnover and microbial mineralization than with uptake alone. Third, the inter-row gradients: pH, AP and OM changed continuously from the control to the inter-row non-rhizosphere soil and then to the rhizosphere soil, and because the inter-row soil is only weakly affected by direct root uptake, its changes likely reflect root penetration, shading with moisture conservation, outward diffusion of exudates and microbial processes; this evidence shows that uptake removal cannot fully explain the soil differences, although it does not exclude an uptake contribution. Consistent with this interpretation, no mowing, harvesting, incorporation, topdressing, or pesticide application was performed during the sampling period, and management was identical across the four green manures and the control (Section 4.1), so differences in aboveground removal cannot explain the observed soil differences.

Legumes obtain nitrogen via biological fixation (acquisitive strategy), with rhizosphere N surplus spilling to inter-row soil [25,40]. Grasses depend on soil mineral N, with high carbon/acid secretion accelerating OM mineralization [41,42] (conservative strategy), for which rhizosphere N depletion is the metabolic cost [20,43]. Configuration should emphasize functional complementarity over single-index ranking [44,45,46].

Integrating REI, PCA and chemotypes: *Ms*—balanced with concurrent N and *p* gains and sustained stability; *Lp*—strong extractable P increase but N depletion; *Tr*—pH buffering with balanced N supply; *Bi*—intermediate but most temporally variable. This divergence provides a functional basis for target-oriented configuration.

### 3.3. Temporal Dynamics and Metabolic Strategies

Cross-stage covariation was generally weak after CLR correction. Amino acids and terpenoids-steroids showed positive covariation with TN-REI in the first cross-stage window (*r* = +0.61 and +0.62, uncorrected *p* < 0.05), but none survived multiple-testing correction. Small polar molecules showed only weak negative covariation with AN-REI. *Ms* exemplifies a high-inertia strategy (smallest exudate fluctuation), aligning with its acquisitive N economy; *Bi* exemplifies a low-inertia strategy (strong temporal restructuring), possibly reflecting a carbon-acid trade-off. High-inertia species suit long-term fertility building [19,20]; low-inertia species may suit stage-specific functional switching. These interpretations remain observational.

### 3.4. Application Constraints and Recommendations

Green manure effects must be evaluated in a tree-green manure binary system [1,2,7]. *Ms*’s rhizosphere N surplus may benefit pear trees directly. *Lp*’s increase in extractable P is accompanied by N depletion, which may intensify competition if overlapping with tree roots [8]. *Tr* offers pH buffering and shallow roots, giving high niche separation from deep pear roots—a safe choice for arid orchards [47].

The comprehensive evaluation (Section 2.6) ranks *Ms* first across all scenarios, stable under equal weighting and tryptophan exclusion. This is consistent with its N-carbon-enriched chemotype and rhizosphere N surplus. If the main limiting factor shifts, indices and weights must be reconstructed. All analyses remain observational. Causal inference requires simulated exudate addition in controlled experiments.

In summary, under N-limited conditions, sole *Ms* sowing is a tentative priority recommendation that requires validation in controlled causal experiments. *Tr* suits alkaline soil pH adjustment and balanced supply. Given universal rhizosphere K depletion, potassium budgets over the whole growing period deserve attention. Follow-up studies should use exogenous addition experiments to establish a causal framework.

More broadly, our results caution against over-interpreting single-molecule correlations without CLR correction or family-level control. The chemotype–soil association is better viewed as a family-structured multivariate trait syndrome than as a list of validated marker compounds. This reframing prioritizes trait-based screening over molecule-centric hypothesis testing in green manure selection.

Several limitations of this study should be made explicit. First, the sampling window covered only a few weeks within a single growing season at one site. The four green manures are perennials, and their full life history strategies, multi-year root turnover, and long-term soil improvement effects were not covered; moreover, the present design cannot distinguish short-term phenological fluctuations among BBCH stages from genuine seasonal effects. Accordingly, three classes of conclusions should be distinguished: inter-specific differences—the family-structured divergence of exudate chemotypes among the four species—are the main conclusions of this study; within-species variation across sampling stages refers only to short-term fluctuations observed between BBCH stages and cannot be interpreted as a seasonal effect; and all inferences about temporal dynamics are restricted to the four sampling points of this study and cannot be extrapolated to a full growing season or to multi-year scales. The chemotype–soil associations reported here should therefore be regarded as exploratory, species-level associations within a single-season window rather than generally applicable causal conclusions.

Second, excavation, rinsing and transfer inevitably impose mechanical and physiological disturbance on the roots. Although the 24 h pre-cultivation solution was discarded to remove wound-induced exudates, this duration was not independently validated by a time series experiment, so the measured exudate profiles may still deviate from those of fully undisturbed field roots; future work should compare different pre-cultivation durations in time series and less disruptive techniques such as in situ microdialysis [48] in parallel. Third, because exudate collection used a destructive design with independent plants at each sampling, roots were not weighed after collection, and the semi-quantitative indices in Section 2.6 were based on absolute concentrations in the collection solution (mg L^−1^) without normalization to root mass. We note, however, that the main compositional analyses are based on relative abundances (CLR-transformed), and that the first-place ranking of *Ms* is stable across the two-index core framework excluding tryptophan, the three-index equal-weight sensitivity analysis and both evaluation algorithms (TOPSIS and GRA), so this constraint is unlikely to alter the main direction of the inter-specific comparison; future studies should record root fresh or dry mass and normalize exudate concentrations accordingly, to further eliminate the influence of inter-specific root-size differences on the detectability of low-abundance compounds. Finally, this study included only two species each from the legume and grass families; the observed chemotype–soil coupling therefore reflects family-level differences among the four studied species and cannot be taken as evidence of a general phylogenetic signal, which would require independent sampling across more families and more species.

In addition, several plant-side variables were not measured. Aboveground biomass and yield were not determined because the destructive exudate collection design required intact root systems and no yield-sampling quadrats were established in parallel; the plant density reported in Section 4.1 serves only as an indirect reference of stand establishment and growth vigor and cannot substitute for biomass or yield. Plant tissue N, P, and K concentrations were likewise not measured, so nutrient removal budgets cannot be calculated, and the relative contributions of root uptake and root exudation to the observed soil differences cannot be fully separated. Root length density, root surface area, root dry weight and the root-to-shoot ratio were not determined either, which further limits the separation of root mass effects from exudation effects. Inter-specific differences in planting density, biomass, root quantity and uptake capacity may therefore confound part of the soil property variation reported here, and the published biomass and nutrient uptake data of orchard green manures [3,5,6] provide background reference only and cannot substitute for measurements in this study. It should also be noted that the first-place ranking of *Ms* in Section 2.6 is a relative ordering that is robust to index composition, weighting scheme, and algorithm choice and does not depend on absolute flux normalization, but this robustness cannot replace absolute flux measurement. Future work should couple aboveground and belowground biomass determination, plant tissue nutrient analysis, destructive root sampling and root scanning with nutrient mass-balance calculation—using hydroponic systems or in situ microdialysis [48] to obtain exudation fluxes per unit root length or root surface area—so that exudation effects, root uptake effects, and biomass effects can be quantitatively distinguished.

A further limitation concerns possible microbial turnover during the 48 h exudate collection. Despite the antimicrobial measures described in Section 4.3 (autoclaved pebbles, ultrapure water and Parafilm sealing), microbial transformation of the released compounds cannot be fully excluded, and some detected compounds may therefore partly reflect microbial processing rather than direct root release. Root reabsorption (backflow) of reabsorption-sensitive compounds such as amino acids and sugars, together with adsorption and desorption at root, device and pebble surfaces, may likewise have altered compound concentrations during the 48 h collection and can cause underestimation or overestimation in either direction; the 48 h collection therefore yielded the net root-derived metabolites collected under this experimental protocol, rather than the total amounts exuded. Three considerations nevertheless indicate that this constraint is unlikely to generate the inter-specific differences reported here. First, all species were collected from the same soil, at the same time and with the same device and procedure, so any microbial consumption constitutes a shared, non-species-specific bias rather than a differential treatment effect. Second, the main compositional analyses are based on CLR-transformed relative abundances, which are robust to multiplicative distortions of absolute fluxes such as a common fractional consumption. Third, the chemotype differences are large and consistent across all four stages (Section 2.1), whereas microbial consumption would be expected to blur, rather than create, such structured and temporally coherent differences. Nevertheless, because the four species may differ in rhizosphere microbial activity, reabsorption capacity, and root status, these biases cannot be assumed to be quantitatively identical across species, and the significant Mantel coupling reported in Section 2.5 is consistent with the collected data but does not constitute independent evidence that the collection system is unbiased. Future work should verify this directly through shorter collection windows (2–6 h), in situ microdialysis [48], sterile controls, microbial inhibitors, and ^13^C stable-isotope probing of the collection solution.

## 4. Materials and Methods

### 4.1. Experimental Design and Soil Sampling

The field trial was conducted during the summer growing season (June to July) at the Korla Fragrant Pear Research Centre in Korla, Xinjiang. The pear trees were 8 years old, with 3 m × 3.7 m spacing. During the experimental period, the pear trees were at the fruit development stage (BBCH 71–79 [49]): locally, full bloom of Korla fragrant pear usually occurs on 12–18 April, the fruit development period lasts about 140–150 days and maturity falls in late August to mid-September, so the four sampling dates corresponded to approximately BBCH 71–73 (14 June), BBCH 73–75 (28 June), BBCH 75–77 (12 July) and BBCH 77–79 (25 July). Drip irrigation was applied throughout the trial, with the irrigation amount adjusted to soil moisture and precipitation so that the water content of the plough layer was maintained at about 65% of field capacity; a compound fertilizer (N-P_2_O_5_-K_2_O = 15-15-15) was incorporated once as basal fertilizer at 600–750 kg ha^−1^ before sowing; and no inter-row tillage was performed during the experiment to preserve the natural state of the green manure roots and soil structure. These management measures were identical across the four green manure treatments and the clean-tilled control. No other inter-row operations (such as mowing, topdressing, or pesticide application) were performed during the sampling period, so that the treatments differed only in the green manure species. The four green manures—alfalfa (*Medicago sativa* L., *Ms*), white clover (*Trifolium repens* L., *Tr*), smooth bromegrass (*Bromus inermis* Leyss., *Bi*) and perennial ryegrass (*Lolium perenne* L., *Lp*)—were sown in the inter-rows on 16 April of the experimental year by drill seeding at a depth of 2–3 cm, in a completely randomized block design with four treatments and three replicate blocks. Each green manure plot was 3.7 m × 100 m, equal to a single inter-row spacing. A clean-tilled bare-soil control (CK) was included. After emergence had stabilized, plants of uniform vigor and without visible abnormalities were selected as fixed monitoring plants in each plot. Plant density, measured in 20 cm × 20 cm quadrats, was 5, 4, 3, and 7 plants per quadrat for *Ms*, *Bi*, *Tr,* and *Lp*, respectively. Plant density is reported only as an indirect reference of stand establishment and growth vigor and cannot substitute for aboveground biomass or yield, which were not measured in this study; root length density, root surface area, and root dry weight were likewise not determined, because the destructive root coring and root scanning required for such measurements are incompatible with the shake-root rhizosphere sampling and the in situ exudate collection used here.

Soil was sampled at four key phenological stages: the first growth stage (14 June), the second growth stage (28 June), the first maturation stage (12 July) and the second maturation stage (25 July). Phenological stages at each sampling date were defined according to the BBCH scale [49]. Because the four species differ in life history, their phenology was not fully synchronous, and their developmental states are therefore described per species rather than being forced into a uniform BBCH interval. The erect-stemmed grasses *Bi* and *Lp* were at stem elongation (BBCH 31–39) on 14 June, at heading (BBCH 51–59) on 28 June, at flowering (BBCH 61–69) on 12 July, and at grain filling to the dough stage (BBCH 71–85) on 25 July. The erect-stemmed legume *Ms* was at stem elongation (BBCH 31–39) on 14 June, at budding (BBCH 51–59) on 28 June, at the beginning-to-full flowering stage (BBCH 61–65) on 12 July, and at full flowering to early pod development (BBCH 65–75) on 25 July. The stoloniferous legume *Tr*, sown in the same year, remained in the establishment phase throughout the sampling period, dominated by rosette leaf development and initial stolon extension (BBCH principal stages 1–3), and did not enter reproductive growth (principal stages 5–9), so reproductive-stage codes are not used for this species. These BBCH codes reflect the actual developmental state of each species on each sampling date rather than a forcibly aligned common stage, and the asynchrony among species is part of their biological difference, which the comparisons in this paper acknowledge rather than attempt to eliminate. This window covers only the first growing season after establishment and cannot substitute for observations of complete life histories or multi-year successional effects. In each plot, green manure rhizosphere soil was collected by the shake-root method (soil attached to the root surface at approximately 1–4 mm). The steepest gradients of most rhizosphere parameters occur within 1–2 mm of the root surface [18], so the 1–4 mm layer collected here comprises the rhizosphere sensu stricto plus a narrow transition zone, and each measurement is a spatial average across this layer that may underestimate the modification intensity at the root surface itself; because the identical operational definition was applied to all four species and all batches, inter-specific comparisons remain internally consistent. Inter-row non-rhizosphere soil of the same plot was sampled from the 0–20 cm surface layer by the five-point sampling method and thoroughly mixed into one composite sample representing the soil background of the green manure planting zone. CK soil was sampled from the same layer of the clean-tilled bare land. Samples were transported on ice, sieved (<2 mm), and split for physicochemical and metabolomic analyses. Rhizosphere soil targeted the 1–4 mm microdomain around roots to capture rhizosphere-specific responses, whereas inter-row non-rhizosphere soil and CK soil were composite samples of the 0–20 cm plough layer, consistent with the main root distribution layer of the green manures. This asymmetry has two dimensions. At the interface scale, rhizosphere samples came from the 1–4 mm microdomain adhering to the root surface, because rhizosphere chemical modification occurs only within this thin layer directly influenced by the root, whereas non-rhizosphere background and CK soils were multi-point composite samples of the 0–20 cm inter-row plough layer. At the depth scale, the non-rhizosphere samples covered the 0–20 cm layer uniformly, whereas the depth range of the rhizosphere samples was determined by the actual root distribution and was not uniform through the soil profile. Rhizosphere samples therefore cannot be equated to a uniformly representative sample of the 0–20 cm plough layer, nor can the non-rhizosphere background represent the root surface microdomain. This asymmetric design follows from the research objective itself: rhizosphere sampling targets the chemical microdomain of the root–soil interface, whereas the non-rhizosphere background must characterize the overall condition of the inter-row plough layer. The REI thus describes the additional contribution of the rhizosphere interface relative to the plough layer background, rather than the difference between two parallel scale-matched samples. We explicitly acknowledge that, because the two scales are asymmetric, absolute REI values may be inflated relative to a scale-matched design; the REI was therefore used only to indicate the direction and relative magnitude of rhizosphere versus background differences, not for absolute quantification or cross-study comparison. Within this same asymmetric scheme, however, all four species were sampled at identical scales, so inter-specific REI comparisons remain fair. Scale-matched validation would require additional 1–4 mm non-rhizosphere sampling, which was not included in this study and remains a direction for future improvement.

### 4.2. Soil Physicochemical Analysis

Soil pH was measured in a 1:2.5 (*w*/*v*) soil–water suspension with a glass electrode (pH meter; INESA Scientific Instrument, Shanghai, China). Total nitrogen (TN) was determined by the Kjeldahl method: samples were digested with concentrated H_2_SO_4_ and a mixed catalyst (K_2_SO_4_–CuSO_4_–Se), followed by steam distillation of the released ammonia into boric acid and titration with standard acid. Available phosphorus (AP) was extracted with 0.5 mol L^−1^ NaHCO_3_ (pH 8.5; Olsen method; soil-to-solution ratio 1:20, shaken for 30 min at 25 °C) and determined colorimetrically by the molybdenum–antimony anti-spectrophotometric method at 880 nm using a spectrophotometer (Jinghua Scientific Instrument, Shanghai, China). Organic matter (OM) was determined by the potassium dichromate oxidation method with external heating (K_2_Cr_2_O_7_–concentrated H_2_SO_4_), the excess dichromate being back-titrated with FeSO_4_, and a conversion coefficient of 1.724 was applied. Alkali-hydrolyzable nitrogen (AN) was determined by the alkaline hydrolysis–diffusion method: soil was hydrolyzed with 10 mL of 1.8 mol L^−1^ NaOH in a diffusion dish at 40 °C for 24 h, and the released ammonia was absorbed in boric acid and titrated with 0.01 mol L^−1^ standard acid. Available potassium (AK) was extracted with 1 mol L^−1^ ammonium acetate (NH_4_OAc, pH 7.0; soil-to-solution ratio 1:10, shaken for 15 min) and measured by flame photometry (FP6410 flame photometer; INESA Analytical Instrument, Shanghai, China). Electrical conductivity (EC) was measured at a soil-to-water ratio of 1:5 using a conductivity meter (INESA Scientific Instrument, Shanghai, China). Reagent blanks and standard curves were included in each analytical batch, and three biological replicates were analyzed for each treatment and sampling date. All procedures followed the standard protocols of Bao [50].

### 4.3. Root Exudate Collection and GC-MS Analysis

Root exudates were collected in situ on a solid support [51,52]. Intact root systems of plants within the monitoring quadrats were carefully excavated with minimal mechanical damage, rinsed with deionized water and placed in an in situ collection device (PVC pipe, inner diameter 10 cm, length 21 cm, total volume approximately 1.65 L), in which the root position was fixed with sterile pebbles (1–1.5 cm in diameter, autoclaved at 121 °C for 20 min before use) packed to about one third of the device height, so that the roots were in full contact with the collection solution while maintaining their in situ angle. A 500 mL volume of nutrient solution, sufficient to immerse the root system while leaving headspace and matching the device volume for ease of operation and recovery, was injected into the device, which was then buried back at the original position at an inclination of 60–70° to maintain the in situ root environment. The opening was sealed with Parafilm to exclude soil particles and impurities. After 24 h of pre-cultivation, the collection solution was discarded to eliminate stress effects caused by mechanical damage; the solution was then replaced with fresh carbon-free nutrient solution, and collection continued for 48 h. The two stages differed in solution composition. The pre-cultivation solution contained urea 0.03 g L^−1^, KH_2_PO_4_ 0.014 g L^−1^, K_2_SO_4_ 0.035 g L^−1^, MgSO_4_ 0.02 g L^−1^ and CaCl_2_ 0.03 g L^−1^, with urea retained as a nitrogen source to maintain basic root physiology and to promote recovery from the mechanical stress of excavation and rinsing; after pre-cultivation, the solution was completely discarded, and the inner wall of the device and the root surface were rinsed three times with ultrapure water and then with the carbon-free basal solution, so as to thoroughly remove residual urea and the exudates produced during pre-cultivation. The formal collection stage used a carbon-free basal nutrient solution with the same mineral composition but without urea, so as to avoid interference of exogenous carbon-containing compounds with GC-MS detection. This staged isolation was adopted because wound-induced exudation typically peaks within hours of mechanical damage and then declines; discarding the entire pre-cultivation solution, rather than only the initial fraction of the formal collection, confines the subsequent 48 h collection to a relatively stable exudation period. The 24 h duration follows published in situ collection protocols [51,52] and is a conservative choice within that literature range; however, whether this duration fully eliminates stress effects was not independently validated by a time series experiment in this study, and this constraint is stated in Section 3.4. During formal collection, the solution volume in each device was maintained at 500 mL: whenever the volume decreased through root uptake or evaporation, an equal volume of the same carbon-free basal solution was replenished, which introduced no exogenous carbon, and exudate concentrations were finally calculated on the uniform 500 mL basis. Because the four species differ inherently in root system size—a species trait that cannot be eliminated by manipulation—roots of each species were packed at a reasonable density matched to the internal volume of the device, so that the roots were in full contact with the collection solution without overcrowding. As the destructive design used independent plants at each sampling date, roots were not oven-dried and weighed after collection; exudate indices were therefore not normalized to root mass, by design rather than by operational limitation, and the implications of this constraint are addressed in Section 3.4. At the end of collection, the solution was withdrawn with a vacuum pump, the inner wall of the device and the root surface were rinsed with a small volume of carbon-free nutrient solution, and the rinsate was pooled with the collection solution to obtain the crude exudate. The collection solution was immediately transferred to a vehicle-mounted refrigerator, kept temporarily at 4 °C, transported to the laboratory as soon as possible, aliquoted, and stored at −80 °C pending GC-MS analysis. Microbial growth during collection was minimized by the use of autoclaved pebbles, ultrapure water for all solutions, and the Parafilm seal. These measures reduce, but cannot exclude, microbial turnover of the released compounds over the 48 h collection period; the consequences of this constraint for the inter-specific comparisons are evaluated in Section 3.4.

The crude exudate was freeze-dried (freeze dryer; Haier Biomedical, Qingdao, China) and extracted with 2 mL methanol (chromatographic grade; Macklin, Shanghai, China), 2 mL acetonitrile (Tianjin Zhiyuan Chemical Reagent, Tianjin, China) and 1 mL pyridine (Sinopharm Chemical Reagent, Shanghai, China). The extract was filtered through a 0.22 μm organic membrane to enrich the analytes and remove impurities, blown to dryness under nitrogen (HD-QD12 nitrogen evaporator; Shandong Holder Electronic Technology, Weifang, China) at ambient pressure and 35 °C for 5 min, and then derivatized.

For derivatization, 80 μL of a freshly prepared 30 mg mL^−1^ methoxyamine hydrochloride (Aladdin, Shanghai, China) solution in pyridine was added to the dried sample and vortexed for 1–2 min until fully dissolved (if not dissolved after 1 min of vortexing, sonication for 1 min was used). The mixture was reacted at 40 °C for 60 min in a water bath, with vortexing every 20 min to ensure complete reaction. Then 99 μL of N,O-bis(trimethylsilyl)trifluoroacetamide (BSTFA; Macklin, Shanghai, China) and 1 μL of trimethylchlorosilane (TMCS; Macklin, Shanghai, China) were added directly, vortexed, and reacted at 70 °C for 60 min. After cooling at room temperature for 5 min, the product was transferred to a GC-MS vial with an insert and sealed, and injected within 24 h to avoid hydrolysis of the derivatives.

GC-MS analysis was performed on a GCMS-QP2020 NX system (Shimadzu, Kyoto, Japan) equipped with an Rtx-5MS capillary column [10] (30 m × 0.25 mm × 0.25 μm; Restek, Bellefonte, PA, USA) with high-purity helium as the carrier gas. Splitless injection of 1 μL was used. The oven temperature program was 60 °C held for 2 min, increased at 30 °C min^−1^ to 120 °C, then increased at 5 °C min^−1^ to 330 °C and held for 8 min. Mass spectra were acquired in full-scan mode with an ion source temperature of 230 °C, quadrupole temperature of 150 °C, solvent delay of 5 min, and scan range of *m/z* 50–600.

Compounds were identified by combined library searching against the NIST 20 and GMD libraries with deconvolution in AMDIS (version 2.73, NIST, Gaithersburg, MD, USA) [53,54]. Putative identifications were manually verified using retention index (RI) deviation within ±30 and mass spectral consistency. For candidate peaks exhibiting low library match scores or RI deviations beyond the tolerance threshold, additional manual interrogation was performed based on diagnostic fragment ions and fragmentation pathways under electron impact ionization. Characteristic low-mass fragments and neutral loss sequences (e.g., those indicative of sugars and their derivatives) were extracted to locate and confirm chromatographic peaks from co-eluting or low signal-to-noise backgrounds, aided by retention time windows and elution order of homologous compounds. Column bleed and environmental contaminants were excluded based on diagnostic ion pairs. Only target root exudate compounds that passed this comprehensive confirmation workflow were retained. Relative contents were calculated by peak area normalization.

For semi-quantification, norvaline (Macklin, Shanghai, China) was used as the internal standard at a working concentration of 4 mg L^−1^. Multiple chromatographic peaks of the same compound within one sample were first summed. The semi-quantitative concentration was calculated as semi-quantitative concentration (mg L^−1^) = (summed peak area of the compound/peak area of norvaline) × 4, assuming a relative response factor of unity for every compound against the internal standard; compounds not detected were scored as zero. Semi-quantification with a single internal standard rather than absolute quantification with standard curves was chosen because most amino acids and nitrogen-containing compounds in root exudates lack isotope-labeled internal standards, and derivatization response factors reported in the literature vary widely (approximately 0.4–1.3) and are mostly derived from non-identical matrix conditions; applying such factors would introduce additional uncertainty. Where response factors are unknown but expected to be similar, assigning a uniform response factor of one provides a consistent, transparent, and comparable flux scale for inter-specific comparison. Semi-quantification focused on 21 amino acids and nitrogen-containing compounds in the two maturation stage batches (12 and 25 July). The identical collection device, nutrient solution volume, and collection duration applied to all species and batches provided a consistent operational basis, so the semi-quantitative concentrations are relatively comparable across species; however, because they were not normalized to root length, root surface area, or root dry weight, they cannot be interpreted as absolute exudation fluxes (e.g., in mol d^−1^ cm^−1^ root length or mol d^−1^ cm^−2^ root surface area).

### 4.4. Statistical and Compositional Data Analysis

The 112 compounds were assigned to seven classes according to their chemical structure and previously proposed rhizosphere functions: sugars and sugar alcohols; amino acids and nitrogen-containing compounds; small polar molecules; phenolics and aromatics; terpenoids, steroids and lipophilic signal molecules; long-chain aliphatic compounds; and others. Class relative abundances were first summed across compounds within each biological replicate and then averaged across replicates with standard deviations; compositional figures were normalized to 100% per batch.

Compositional data analysis. Because relative abundances are subject to the sum constraint (closure), their direct use in correlation and distance analysis generates spurious correlations; all associations between exudate composition and soil indices were therefore analyzed from raw peak areas using a compositional data workflow. Multiple peak records of the same compound within the same biological replicate were first summed and normalized to the total peak area of the sample. Zeros were replaced by one half of the minimum positive relative abundance within each sample (multiplicative replacement strategy for compositional data [30,31,55]), the data were reclosed, and a center log-ratio (CLR) transformation was applied. Between-sample compositional differences were measured as Euclidean distances of CLR coordinates (Aitchison distances).

Multivariate analysis and hypothesis testing. All statistical analyses were performed in R (version 4.5.2, R Foundation for Statistical Computing, Vienna, Austria); two-way ANOVA with Tukey’s HSD test, PCA, Spearman correlation and Benjamini–Hochberg adjustment were computed with the built-in stats package, PERMANOVA and the Mantel and partial Mantel tests with the vegan and ecodist packages, and entropy weighting, TOPSIS and GRA with custom R scripts. Exudate classes and soil indices were analyzed by two-way analysis of variance (species × stage) with Tukey’s honestly significant difference test (*p* < 0.05). Principal component analysis (PCA) was applied to the standardized rhizosphere soil property matrix including CK as a reference, and permutational multivariate analysis of variance (PERMANOVA, 999 permutations) was used to test the significance of species clustering (CK excluded). Mantel correlation (Pearson, 999 permutations) between the overall exudate composition (Aitchison distance of CLR coordinates) and the standardized soil property matrix (Euclidean distance) was computed from species–stage centroids (4 species × 4 stages, *n* = 16; each centroid being the mean of three biological replicates), with a partial Mantel test controlling for family. Associations of class-level and single-molecule CLR coordinates with individual soil indices were tested using Spearman correlation. For class-level CLR coordinates, the seven class relative abundances were treated as a seven-part composition; zeros were replaced and CLR-transformed following the same procedure as for the overall composition (Figure 5b). All 784 compound–index tests for single-molecule screening were adjusted by the Benjamini–Hochberg method at a false discovery rate [32]. Centroid-level analyses used the 16 combination means as units; replicate-level analyses used the original 48 samples (4 species × 4 stages × 3 replicates). The partial Mantel test and cross-stage covariation analysis were based on REI to remove background variation of inter-row non-rhizosphere soil. The REI matrix used for the Mantel test was column-wise *z*-standardized before Euclidean distances were computed, so that differences in scale would not allow absolute difference indices such as pH to be overwhelmed by percentage-type indices. The cross-stage covariation analysis used the interval between adjacent samplings (approximately 13–14 days) as one cross-stage unit and computed Spearman correlations between exudate classes at stage T and soil REI at stage T + 1, with the correlation at stage T as the concurrent control (concurrent *n* = 16; cross-stage pairs *n* = 12). This cross-stage window was defined by the field sampling design rather than by the precise time scale of rhizosphere process kinetics, and the results should be regarded as a short-term exploratory trend across sampling stages rather than confirmatory causal inference. For missing values, compounds not detected (recorded as ‘-‘) were treated as zero in class summation; multiple comparisons were performed only among detected treatment combinations. Compounds detected in only one stage were used solely to describe species specificity. All analyses were performed in Python 3.12 with scikit-learn, scipy, and numpy.

Comprehensive evaluation. Four green manures were evaluated using three positive indices constructed from maturation stage semi-quantification: the abundance of amino acids and nitrogen-containing compounds (mean of the two batches, mg L^−1^), tryptophan semi-quantitative concentration (mean of the two batches, mg L^−1^), and a temporal stability index, Tstab = 1 − |mean at 25 July − mean at 12 July|/(mean at 25 July + mean at 12 July), where means are class abundance batch means; values closer to one indicate smaller across-stage fluctuation. Index weights were determined by the entropy method [56]: p_ij_ = x_ij_/Σ_i_ x_ij_, e_j_ = −(1/ln 4) Σ_i_ p_ij_ ln p_ij_, and w_j_ = (1 − e_j_)/Σ_j_ (1 − e_j_). TOPSIS (vector normalization followed by weighting [57]; closeness C_i_ = D_i_^−^/(D_i_^+^ + D_i_^−^), where D_i_^+^ and D_i_^−^ are the Euclidean distances to the positive and negative ideal solutions, respectively) and gray relational analysis (GRA; indices were range-normalized [58], the optimal sequence was used as the reference sequence, the distinguishing coefficient ρ was set to 0.5, and the relational grade ξ_i_ = Σ_j_ w_j_ ξ_ij_) were used to compute composite scores and rankings, and an equal-weight scenario (*w* = 1/3) was used as a sensitivity test.

## 5. Conclusions

The four green manures formed distinguishable exudate chemotypes with species-specific temporal fluctuation, establishing that the temporal inertia of exudate composition is itself a quantifiable functional trait. Rhizosphere effects diverged systematically: grasses showed the strongest increases in extractable P at the cost of rhizosphere nitrogen consumption, whereas legumes accumulated nitrogen efficiently and spilled the surplus into inter-row soil, with potassium depleted across all species. Green manure improvement followed a two-layer structure: background elevation in inter-row soil superimposed with rhizosphere hot-spot intensification.

The chemotype–soil coupling, after CLR correction, was largely explained by family-level phylogenetic background: the partial Mantel correlation fell from 0.246 to 0.062 when family was controlled. Class-level signals, particularly of amino acids, constituted the main tier associated with nitrogen-cycle improvement, whereas single-molecule correlations served only as exploratory indicators, did not survive multiple-testing correction, and were statistically indistinguishable from family-level background. Most species-specific high-abundance compounds therefore function primarily as chemotype identity markers rather than causal drivers.

Functionally, Ms is multifunctional and balanced; Lp excels in increasing extractable P but depletes rhizosphere nitrogen; Tr offers clear advantages in pH buffering and niche separation; and Bi is less predictable. Nitrogen-anchored evaluation ranked Ms first, supported by its N-enriched chemotype and measured rhizosphere N surplus. The exploratory tryptophan correlation, nested as it is within the family-level background, is treated only as corroborative background for this ranking, not as independent mechanistic evidence. Given the universal rhizosphere potassium depletion, potassium budgets over the whole growing period deserve attention in orchard management. In summary, our results demonstrate that the chemotype–soil association is better understood as a family-structured multivariate trait syndrome than as a list of validated marker compounds. This finding carries three implications. Methodologically, it underscores that CLR correction and family-level control are mandatory before any single-molecule inference can be drawn from root exudate profiles. Theoretically, it suggests that, in the four species studied, rhizosphere functional differentiation among green manures is largely structured along the legume–grass family divide, with ontogenetic fine-tuning playing a secondary role. Practically, the nitrogen-anchored multi-criteria evaluation (entropy-weighted TOPSIS and GRA) provides a transferable pipeline for functional screening of green manures, moving beyond molecule-centric hypothesis testing toward trait-based selection. This ranking applies specifically to nitrogen-limited soils; if increasing extractable P becomes the management objective, the priority of Lp rises accordingly. Field configuration should follow target-oriented selection based on site-specific limiting factors, and causal inference requires verification by independent controlled experiments. These findings derive from a few weeks of a single growing season at one site and are therefore exploratory; the chemotype–soil associations reported here should be regarded as hypothesis-generating patterns, whose causal resolution awaits family-level sampling designs combined with microbiome manipulation experiments, and multi-year, multi-site experiments across full phenological cycles are needed before these associations can be generalized.

## Figures and Tables

**Table 1:** Soil physicochemical properties under different treatments.

Treatment	Soil Type	pH	TN (g kg^−1^)	AN (mg kg^−1^)	AP (mg kg^−1^)	OM (g kg^−1^)	AK (mg kg^−1^)	EC (dS m^−1^)
CK	Non-rhizosphere (0–20 cm)	8.52 ± 0.08	0.836 ± 0.037	17.4 ± 1.1	24.8 ± 2.2	16.6 ± 1.1	170.3 ± 8.2	1.41 ± 0.04
*Ms*	Non-rhizosphere (0–20 cm)	8.33 ± 0.05	0.895 ± 0.065	21.0 ± 1.3	38.2 ± 2.9	19.2 ± 1.2	177.2 ± 7.6	1.39 ± 0.05
*Ms*	Rhizosphere (1–4 mm)	8.31 ± 0.11	1.071 ± 0.101	24.9 ± 1.7	48.3 ± 7.7	21.3 ± 2.0	168.5 ± 3.8	1.49 ± 0.06
*Bi*	Non-rhizosphere (0–20 cm)	8.36 ± 0.12	0.882 ± 0.036	18.9 ± 1.8	33.6 ± 5.3	18.8 ± 1.9	175.5 ± 6.7	1.36 ± 0.05
*Bi*	Rhizosphere (1–4 mm)	8.34 ± 0.16	0.827 ± 0.034	18.4 ± 1.8	38.1 ± 6.7	20.6 ± 1.0	166.5 ± 7.1	1.41 ± 0.08
*Tr*	Non-rhizosphere (0–20 cm)	8.41 ± 0.09	0.924 ± 0.058	20.1 ± 1.7	33.3 ± 3.2	17.8 ± 1.6	176.4 ± 16.8	1.37 ± 0.06
*Tr*	Rhizosphere (1–4 mm)	8.37 ± 0.12	1.077 ± 0.102	23.0 ± 2.8	39.2 ± 2.7	19.0 ± 1.4	170.5 ± 10.1	1.45 ± 0.04
*Lp*	Non-rhizosphere (0–20 cm)	8.34 ± 0.07	0.878 ± 0.063	18.7 ± 1.2	32.8 ± 3.6	19.9 ± 1.5	177.7 ± 8.3	1.41 ± 0.07
*Lp*	Rhizosphere (1–4 mm)	8.29 ± 0.13	0.829 ± 0.052	16.5 ± 1.6	44.9 ± 7.1	22.4 ± 2.8	165.1 ± 5.7	1.52 ± 0.07

Note: Data are presented as means ± SD. TN, total nitrogen; AN, alkali-hydrolyzable nitrogen; AP, available phosphorus; OM, organic matter; AK, available potassium; EC, electrical conductivity. The rhizosphere samples (soil adhering to the root surface within approximately 1–4 mm) include the rhizosphere sensu stricto plus a narrow transition zone, because the steepest gradients of most parameters occur within 1–2 mm of the root surface [18]; REI values derived from these data are therefore spatial averages at the interface scale.

**Table 2:** Semi-quantitative evaluation indices and composite scores of the four green manures for amino acids and nitrogen-containing compounds at the maturation stage (entropy weighting; three-index sensitivity scenario).

Green Manure	Class Abundance (mg L^−1^)	Tryptophan (mg L^−1^)	T_stab_	TOPSIS C_i_	GRA ξ_i_	Overall Rank
Alfalfa (*Ms*)	29.44	11.16	0.949	0.9995	0.9959	1
White clover (*Tr*)	21.37	7.65	0.939	0.6187	0.5608	2
Smooth bromegrass (*Bi*)	22.38	2.45	0.979	0.0533	0.3590	3
Perennial ryegrass (*Lp*)	20.99	1.96	0.764	0.0000	0.3333	4

## Data Availability

The data presented in this study are available in the article. Raw data are available from the corresponding authors upon reasonable request.

## Supplementary Materials

The following supporting information can be downloaded at: https://www.mdpi.com/article/10.3390/plants15203190/s1, Figure S1: Replicate-level (*n* = 48) correlations of tryptophan with total nitrogen (a) and available nitrogen (b) in rhizosphere soil; Figure S2: Correlations of representative single compounds with rhizosphere soil properties at species × stage centroids (*n* = 16) and within Lp (panel g, *n* = 12).

## Abbreviations

The following abbreviations are used in this manuscript:

PERMANOVA	Permutational multivariate analysis of variance
REI	Rhizosphere effect index
FDR	False discovery rate
CLR	Center log-ratio
PCA	Principal component analysis
BH	Benjamini–Hochberg
CK	Clean-tilled control
BSTFA	N,O-bis(trimethylsilyl)trifluoroacetamide
AMDIS	Automated Mass Spectral Deconvolution and Identification System
GMD	Golm Metabolome Database
TMCS	Trimethylchlorosilane
TOPSIS	Technique for order preference by similarity to ideal solution
