# Supplementary material for: Root Exudate Chemotypes and Soil Improvement in Orchard Green Manures: Species-Specific Associations Reflecting Family-Level Phylogenetic Differences
Source: Plants (Basel). 2026 Oct 18;15(20):3190. doi: 10.3390/plants15203190 (PMC13709027; doi:10.3390/plants15203190)

Figure S1. Replicate-level (n = 48) correlations of tryptophan with total nitrogen (a) and available nitrogen (b) in rhizosphere soil.

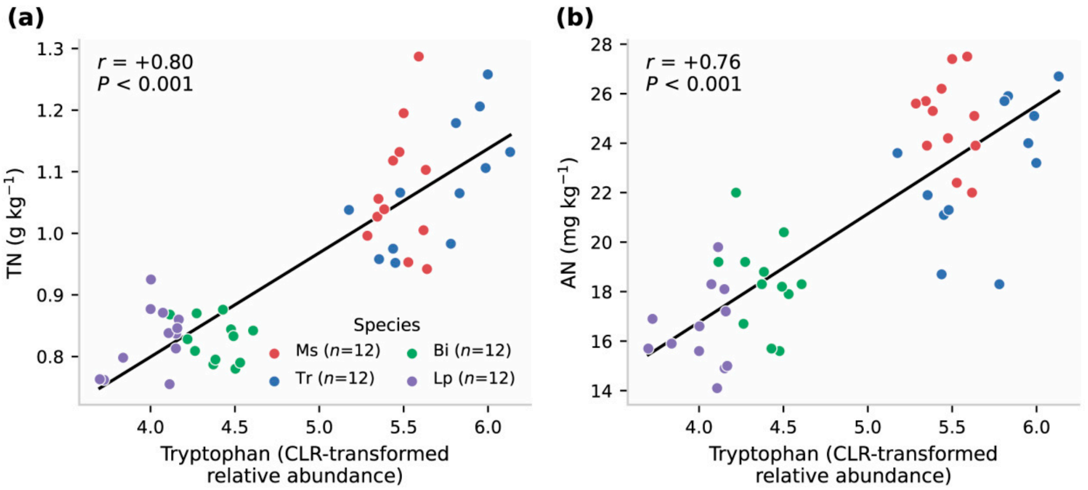

Figure S2. Correlations of representative single compounds with rhizosphere soil properties at species × stage centroids (n = 16) and within Lp (panel g, n = 12).

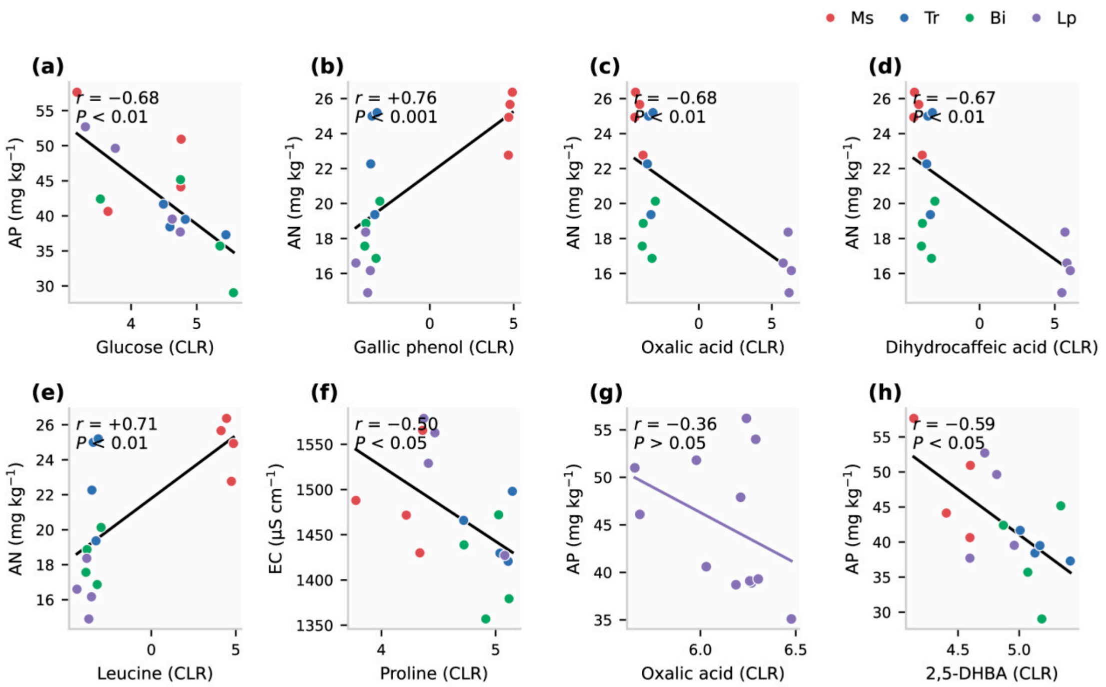

Supplement: Supplementary file 1 [file plants-15-03190-s001.zip › plants-4582930-supplementary.pdf]
